# Endophytic *Trichoderma* strains isolated from forest species of the Cerrado-Caatinga ecotone are potential biocontrol agents against crop pathogenic fungi

**DOI:** 10.1371/journal.pone.0265824

**Published:** 2022-04-15

**Authors:** Estefenson Marques Morais, Alex Ap. Rosini Silva, Francisco Wderson Araújo de Sousa, Izabelle Maria Barboza de Azevedo, Helane França Silva, Alice Maria Gonçalves Santos, José Evando Aguiar Beserra Júnior, Caroline Pais de Carvalho, Marcos Nogueira Eberlin, Andreia M. Porcari, Francisca Diana da Silva Araújo

**Affiliations:** 1 *Campus* Professora Cinobelina Elvas, Federal University of Piauí, Bom Jesus, PI, Brazil; 2 MS4Life Laboratory of Mass Spectrometry, Health Sciences Postgraduate Program, São Francisco University, Braganca Paulista, São Paulo, SP, Brazil; 3 Department of Plant Sciences, *Campus* Ministro Petrônio Portela, Federal University of Piauí, Teresina, PI, Brazil; 4 School of Material Engineering an Nanotechnology, MackMass Laboratory, Mackenzie Presbyterian University, São Paulo, SP, Brazil; Tocklai Tea Research Institute, INDIA

## Abstract

The indiscriminate use of chemical pesticides increasingly harms the health of living beings and the environment. Thus, biological control carried out by microorganisms has gained prominence, since it consists of an environmentally friendly alternative to the use of pesticides for controlling plant diseases. Herein, we evaluated the potential role of endophytic *Trichoderma* strains isolated from forest species of the Cerrado-Caatinga ecotone as biological control agents of crop pathogenic fungi. Nineteen *Trichoderma* strains were used to assess the antagonistic activity by *in vitro* bioassays against the plant pathogens *Colletotrichum truncatum*, *Lasiodiplodia theobromae*, *Macrophomina phaseolina*, and *Sclerotium delphinii* isolated from soybean, cacao, fava bean, and black pepper crops, respectively. All *Trichoderma* strains demonstrated inhibitory activity on pathogen mycelial growth, with maximum percent inhibition of 70% against *C*. *truncatum*, 78% against *L*. *theobromae*, 78% against *M*. *phaseolina*, and 69% against *S*. *delphinii*. Crude methanol extracts (0.5 to 2.0 mg mL^-1^) of *Trichoderma* strains were able to inhibit the growth of *C*. *truncatum*, except *Trichoderma* sp. T3 (UFPIT06) and *T*. *orientale* (UFPIT09 and UFPIT17) at 0.5 mg mL^-1^, indicating that the endophytes employ a biocontrol mechanism related to antibiosis, together with multiple mechanisms. Discriminant metabolites of *Trichoderma* extracts were unveiled by liquid chromatography-tandem mass spectrometry-based metabolomics combined with principal component analysis (PCA), which included antifungal metabolites and molecules with other bioactivities. These results highlight the biocontrol potential of *Trichoderma* strains isolated from the Cerrado-Caatinga ecotone against crop pathogenic fungi, providing support for ongoing research on disease control in agriculture.

## Introduction

Diseases caused by fungi are among the most harmful to plants due to their rapid spread and the ability to adapt to various environmental conditions [1]. The most commonly used methods for the control of plant diseases caused by fungi include the use of chemical fungicides [1, 2], which have disadvantages such as the potential risk of soil and water contamination, damage to human health, and the development of resistance against fungicides by plant pathogens [2, 3].

Research on alternative methods of controlling fungal diseases has been widely developed, often requiring the integrated implementation of several methods, known as integrated disease management (IDM) [2, 4]. Among these methods are the use of disease-resistant cultivars, adequate water and soil management, fertilization, crop rotations, and biological control agents (BCA), aiming to maintain or increase agricultural production with a reduced application of chemical agents [5]. Many studies still need to be developed for the use of biological control of plant pathogens on a global scale, since biopesticides represent only approximately 2% of all pesticides sold in the world [6, 7].

A promising alternative method for plant pathogen control is based on the use of antagonistic microorganisms, such as endophytic fungi, capable of protecting their hosts from the action of pathogens [8, 9]. These microorganisms live inside plant tissues without causing damage in a complex mutualistic relationship, where endophytes receive nutrients and protection, while plants have advantages, such as greater resistance in environments with intense stress caused by biotic (insects, herbivores, nematodes, and phytopathogenic microorganisms) or abiotic factors (pH, temperature, drought and saline stresses, etc.) [10, 11].

Recent research has revealed that although endophytic microorganisms have received global interest, there are still several gaps in knowledge, such as the different biomes explored [12]. Considering that endophytes depend on host species and environmental conditions, the diversity of biomes and endemic plants found in Brazil represents a potential source of new beneficial microbial resources [13]. In this context, the Cerrado-Caatinga ecotone stands out [14], which corresponds to the transition area where ecological communities or ecosystems from the Cerrado and Caatinga biomes coincide [15].

The Cerrado and Caatinga biomes are recognized for their great importance. The Cerrado, also known as the Brazilian savanna, is one of the 25 biodiversity hotspots for conservation priorities in the world [16, 17], and the Caatinga is the only uniquely Brazilian biome, in which most of its biological heritage cannot be found anywhere else in the world [18]. The Cerrado-Caatinga ecotone occupies 1.3% of the Brazilian territory, extending over regions of the Piauí, Bahia, and Minas Gerais states [14], and it presents great species richness, whether from the biomes that formed them or endemic species [15]. Few studies have been carried out to explore the biocontrol potential of endophytic fungal biodiversity in this transition zone, which also requires attention due to increasing anthropogenic degradation with the expansion of agricultural production areas [19].

*Trichoderma* species have been tried as BCA and used as an alternative to synthetic pesticides to control a variety of plant diseases [20]. The biocontrol mechanisms of *Trichoderma* are based on the activation of multiple mechanisms, either indirectly, by competing for space and nutrients, promoting plant growth and plant defensive mechanisms, and antibiosis, or directly, by mycoparasitism [21, 22]. They are found in rhizospheric and non-rhizospheric soils, in addition to their endophytic relationships with many plants [22, 23]. Their biodiversity has been extensively investigated in various geographical locations, and their distribution varies with ecosystems [24, 25]. Therefore, it is fundamental to explore the biocontrol potential of *Trichoderma* strains isolated from native areas, since they represent a tool for sustainable food production.

In this study, we investigated the potential role of endophytic *Trichoderma* strains isolated from forest tree species of the Cerrado-Caatinga ecotone [26, 27] as biological control agents of crop pathogenic fungi. First, we evaluated the interaction between endophytes and pathogens by *in vitro* antagonism bioassays. The biocontrol factor related to antibiosis was examined using *in vitro* bioassays with crude methanolic extracts of *Trichoderma* strains and liquid chromatography-tandem mass spectrometry-based metabolomic approaches. Such data will support ongoing research to find new beneficial microbial resources to control plant diseases.

## Material and methods

### Strains and materials

The nineteen *Trichoderma* spp. isolates (S1 Table) were obtained from leaves of forest tree species (S2 Table), located in a Cerrado-Caatinga ecotone in Southwest Piauí, Brazil (8°51′7.48″ S and 44°11′39.95″ W) [26], and maintained in potato dextrose agar (PDA) culture medium (Himedia). This area comprised a fragment of one hectare within the legal reserve [26]. For the identification of the *Trichoderma* isolates, the gene regions for the translation elongation factor (tef1) and the second largest RNA polymerase subunit (rpb2) were amplified and sequenced, and the construction of phylogenetic trees was performed by comparing the sequences available in GenBank (National Center for Biotechnology Information, NCBI) [27].

The UFPIT01, UFPIT09, UFPIT12, UFPIT14, UFPIT15, UFPIT17, and UFPIT18 isolates were previously identified as *T*. *orientale* (Samuels & Petrini) Jaklitsch & Samuels; the UFPI02 isolate was previously identified as *T*. *longibrachiatum* Rifai; and the UFPI03, UFPI07, UFPI10, UFPI16, and UFPI19 isolates were previously identified as *T*. *koningiopsis* Samuels, Carm. Suárez & H.C. Evans [27]. The UFPIT04, UFPIT05, UFPIT06, UFPIT08, UFPIT11, and UFPIT13 isolates were not identified by comparing the sequences available in GenBank and may likely constitute new species; therefore, they were named *Trichoderma* sp. T1, *Trichoderma* sp. T2, *Trichoderma* sp. T3, *Trichoderma* sp. T4, *Trichoderma* sp. T5, and *Trichoderma* sp. T6, respectively [27]. The strains were registered in the National System for the Management of Genetic Heritage and Associated Traditional Knowledge (SisGen) by n° A7580C1 and A1B50F7, as recommended by the Brazilian Biodiversity Law (n° 13.123/15).

For plant pathogenic fungi tested for antagonism bioassays, *Colletotrichum truncatum* (Schwein.) Andrus & W.D. Moore strain was isolated from infected soybean pods, located in the same mesoregion where *Trichoderma* strains were found, through the cultivation of infected material in PDA medium incubated at 25°C under a 12 h photoperiod [28]. The *Lasiodiplodia theobromae* (Pat.) Griffon & Maubl. strain was isolated from cacao fruit with symptoms of Lasiodiplodia canker. *Macrophomina phaseolina* (Tassi) Goid. COUFPI 10 and COUFPI 11 strains were isolated from the seeds and roots of fava bean, respectively, placed in PDA medium and incubated at 25°C for seven days [29]. *Sclerotium delphinii* Welch COUFPI 209 and COUFPI 249 strains were isolated from black pepper with symptoms of concentric leaf spots by inoculating sclerotia in PDA medium [30]. All strains were maintained in PDA culture medium at 28°C in the absence of light and preserved using Castellani’s method.

Liquid chromatography–mass spectrometry (LC–MS)-grade methanol and acetonitrile were purchased from J.T. Baker (Center Valley, PA, USA). Analytical grade formic acid and sodium formate encephalin were purchased from J.T. Baker (Center Valley, PA, USA), and leucine enkephalin from Waters (Manchester, UK).

### *In vitro* antagonism bioassays against plant pathogenic fungi

Culture medium fragments (1 cm^2^) with *Trichoderma* spp. mycelia and fungal plant pathogens (1 cm^2^), previously cultivated in PDA at 28°C, were transferred to PDA medium 5 cm apart from each other [31]. The plates were incubated at 28°C, and mycelial growth was evaluated daily for seven days. The experimental design was completely randomized with 20 treatments and 3 replications, totaling 60 experimental units for the bioassays of each plant pathogen. The treatments consisted of 19 *Trichoderma* isolates plus a control sample containing only the plant pathogen.

The antagonistic potential was measured as the percent inhibition, according to the formula: % inhibition = (DC-DT/DC)*100, where DT is the growth radius of the plant pathogen colony toward the antagonist and DC is the growth radius of the control [32]. The mycelial growth rate index (MGRI) was obtained from the averages of the daily values of mycelial growth for each treatment, according to the formula MGRI = Ʃ(D-Da)/N, where D = current average colony diameter, Da = average colony diameter from the previous day, and N = number of days after inoculation [33]. Analysis of variance followed by the Scott–Knott test at the 5% significance level was conducted using R v.3.5.2 software (R Core Team, Vienna, Austria).

### Inhibitory activity bioassay of *Trichoderma* spp. organic extracts against *C*. *truncatum*

For the extraction of bioactive compounds, *Trichoderma* strains were inoculated on PDA at 28°C in the absence of light for four days. Subsequently, the culture media (60 x 15 mm) containing the fungal colonies were cut into small pieces, and cold methanol (15 mL) was added. The samples were vortexed for 1 min, allowed to rest for 5 min, and vortexed again for 1 min. Subsequently, the extracts were centrifuged at 4,000 g for 15 min at 4°C, and the supernatants were concentrated under a flow of nitrogen gas [34]. Then, the extracts were weighed, and dimethylsulfoxide (DMSO) was added to prepare a 100 mg mL^-1^ stock solution.

Methanolic extracts of *Trichoderma* spp. were used to evaluate the inhibitory activity against *C*. *truncatum*. For this purpose, fragments of the phytopathogen (9 mm^2^) were inoculated in the center of Petri dishes (60×15 mm) containing PDA with increasing concentrations of the extract (0.0, 0.5, 1.0, and 2.0 mg mL^-1^). For the control (0.0 mg mL^-1^), only DMSO was added. Plates were kept in B.O.D. incubator (Bio-Oxygen Demand) at 28°C in the absence of light, and colony diameters were measured daily for 10 days with the aid of a digital caliper. The experimental design was completely randomized with extracts from 19 isolates at concentrations of 0.0, 0.5, 1.0, and 2.0 mg L^-1^, with three replications for each concentration. Data were subjected to analysis of variance, followed by regression analysis using R software and SigmaPlot v11.0 (Systat Software Inc. Chicago, USA). Additionally, Pearson’s correlation analysis of the percent inhibition of *Trichoderma* strains against *C*. *truncatum* in co-culture and crude extract bioassays was performed.

### Metabolic fingerprinting by liquid chromatography–high resolution mass spectrometry

#### Sample preparation

For extract preparation of the 19 *Trichoderma* isolates, the culture media (60 x 15 mm) containing the fungal colonies, previously cultivated on PDA at 28°C for four days, were cut into small pieces and extracted with methanol (15 mL), vortexed for 1 min, maintained at rest for 5 min, and vortexed again for 1 min. After filtration, the supernatants were concentrated to approximately 1 mL, lyophilized, and stored at -47°C until use. For analyses by ultra-high-performance liquid chromatography coupled with electrospray ionization quadrupole time-of-flight mass spectrometry (UPLC-ESI-Q-TOF-MS), lyophilized samples were reconstituted in a solution containing water/methanol/acetonitrile (1:2:2 v/v/v, 1 mL). The samples were vortexed for 1 min, sonicated for 30 min at room temperature, filtered using a 0.22 μm PTFE syringe filter (Millipore, USA), and transferred to vials for LC–MS analysis [35].

#### UHPLC-ESI-Q-TOF-MS analysis

An ACQUITY UPLC connected to a XEVO-G2XS QTOF mass spectrometer (Waters, Manchester, UK) equipped with an electrospray ion source was used. Liquid chromatography was performed using a Titan™ C18 UHPLC column (2.1 x 100 mm, 1.9 μm, Supelco). The column temperature was maintained at 45°C. The separation was performed at a flow rate of 0.4 mL min^-1^ under a gradient program in which the mobile phase consisted of (A) 0.1% formic acid (v/v) and (B) pure methanol. The gradient program was applied as follows (in % B): (t) = 0 min, 1%; t = 2.0 min, 1%; t = 8.0 min, 38%; t = 20 min, 99.5%; t = 25 min, 99.5%; t = 25.1 min, 1%; and t = 28 min, 1%, for a total analysis time of 28 minutes. The injection volume was 0.2 μL. Positive ion mode was recorded, and the instrument was operated in data-independent acquisition mode (MS^E^). The *m/z* range was 100–1700, with an acquisition rate of 0.5 sec per scan. The following instrumental parameters were used: capillary: 3.0 kV; cone: 40,000 V; desolvation temperature: 550°C; cone gas flow: 10 L h^-1^; desolvation gas flow: 900 L h^-1^. The collision energy was 20 to 60 eV for fragmentation. Leucine encephalin (molecular weight = 555.62; 200 pg μL^-1^ in 1:1 acetonitrile:water) was used as the lock mass for accurate mass measurements, and a 0.5 mM sodium formate solution was used for calibration. Samples were randomly analyzed.

#### Data processing and statistical data analysis

LC–MS raw data were processed using Progenesis QI 2.0 software (Nonlinear Dynamics, Newcastle, UK), which enabled the selection of possible adducts, peak alignment, deconvolution, and putative metabolite identification based on MS^E^ experiments. Progenesis QI generates a table of the ions labeled according to their nominal masses and retention times as a function of their intensity for each sample. The MassBank database (https://massbank.eu) and Vaniya/Fiehn Natural Products Library (https://mona.fiehnlab.ucdavis.edu/) were used to perform the identification using the following search parameters: precursor mass error ≤ 5 ppm and fragment tolerance ≤ 10 ppm.

The list of extracted ion chromatograms by retention time was uploaded to the MetaboAnalyst 5.0 web platform (http://www.metaboanalyst.ca) for principal component analysis (PCA). Ions detected in at least 10% of the samples were retained for analysis, and an interquartile range (IQR) filter was used. Data were sum-normalized, and Pareto scaling was used. A heatmap and unsupervised hierarchical clustering were performed using 50 features with the lowest adjusted p value < 0.05 depicting differential peaks.

## Results and discussion

### Endophytic *Trichoderma* strains from forest species inhibit several crop pathogenic fungi

**Fig 1:**
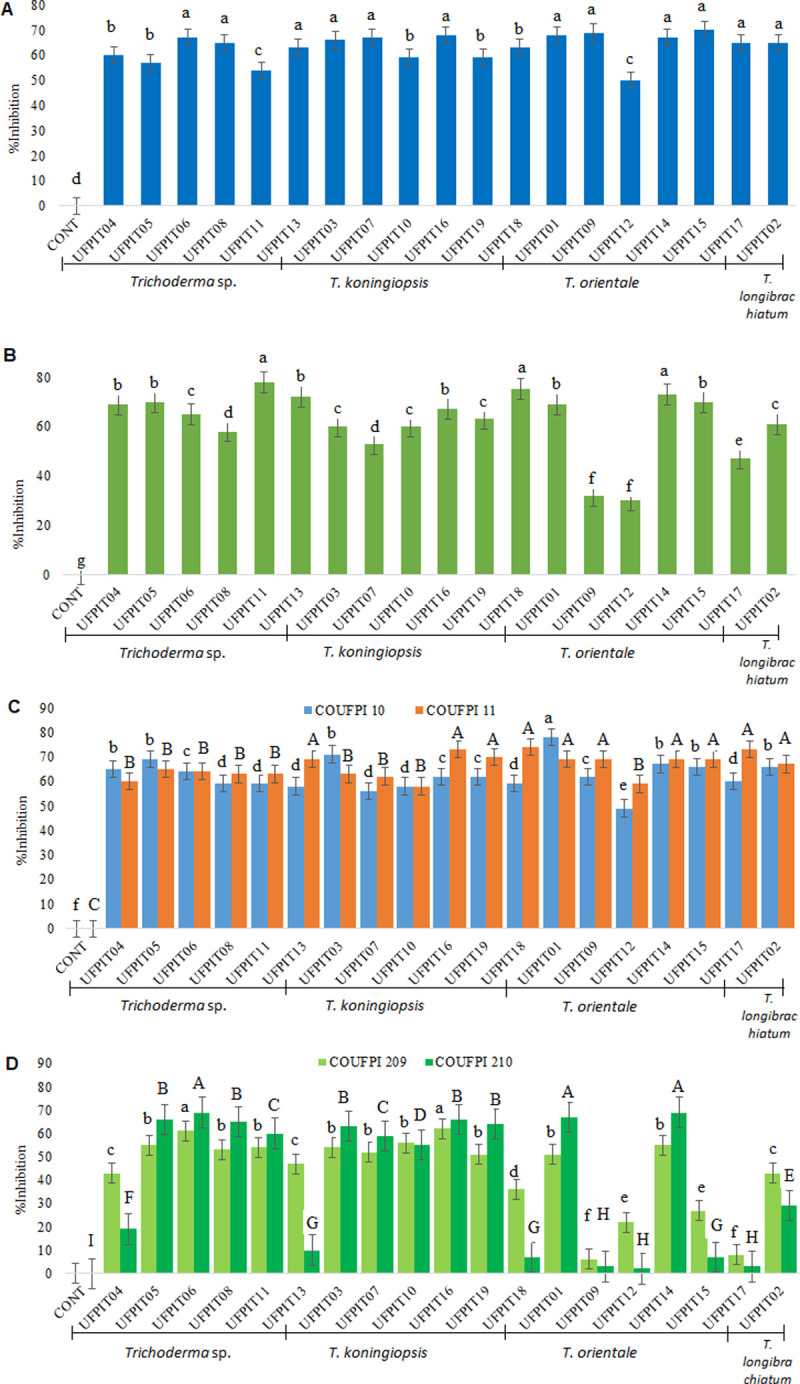
*In vitro* percent inhibition of *Trichoderma* spp. isolates against *C*. *truncatum* (A), *L*. *theobromae* (B), *M*. *phaseolina* COUFPI 10 and COUFPI 11 (C), and *S*. *delphinii* COUFPI 209 and COUFPI 249 (D). Averages followed by the same lowercase or capital letter are not significantly different by the Scott–Knott test at the 5% confidence level. The variation coefficients (CVs) were 4.67% for *L*. *theobromae*, 4.55% for *M*. *phaseolina* COUFPI 10, 5.96% for *M*. *phaseolina* COUFPI 11, 8.37% for *S*. *delphinii* COUFPI 209 and 6.02% for *S*. *delphinii* COUFPI 249.

The antagonistic potential of the endophytic *Trichoderma* spp. strains was investigated against different plant pathogens. The 19 *Trichoderma* spp. isolates demonstrated inhibitory activity against mycelial growth, ranging from 50 to 70% for *C*. *truncatum* (Fig 1A), 30 to 78% for *L*. *theobromae* (Fig 1B), 49 to 78% for *M*. *phaseolina* COUFPI 10 (Fig 1C), 58 to 74% for *M*. *phaseolina* COUFPI 11, 6 to 62% for *S*. *delphinii* COUFPI 209 (Fig 1D), and 2 to 69% for *S*. *delphinii* COUFPI249.

Regarding the inhibition of *C*. *truncatum* (Fig 1A), the fungi *T*. *orientale* (UFPIT01, UFPIT09, UFPIT14, UFPIT15, and UFPIT17), *T*. *longibrachiatum* (UFPIT02), *T*. *koningiopsis* (UFPIT03, UFPIT07, and UFPIT16), *Trichoderma* sp. T3 (UFPIT06), *Trichoderma* sp. T4 (UFPIT08), and *Trichoderma* sp. T6 (UFPIT13) stood out with the highest percent inhibition, from 63 to 70%, and *Trichoderma* sp. T5 (UFPIT11) and *T*. *orientale* (UFPIT12), with the lowest values ranging from 50 to 54%. *Trichoderma* sp. T5 (UFPIT11) and *T*. *orientale* (UFPIT14 and UFPIT18) showed the highest percentage inhibition against *L*. *theobromae* (Fig 1B) of 73–78%, while *T*. *orientale* (UFPIT09 and UFPIT12) had the lowest performance of 30–32%.

Against *M*. *phaseolina* COUFPI 10 (Fig 1C), *T*. *orientale* UFPIT01 and UFPIT12 yielded the highest (78%) and lowest (49%) percent inhibition, respectively, while the other isolates showed intermediate values, above 50% inhibition. *T*. *orientale* (UFPIT01, UFPIT09, UFPIT14, UFPIT15, UFPIT17, UFPIT18), *T*. *longibrachiatum* (UFPIT02), *Trichoderma* sp. T6 (UFPIT13), and *T*. *koningiopsis* (UFPIT16 and UFPIT19) stood out in inhibiting the growth of *M*. *phaseolina* COUFPI 11, from 67 to 74%, and the others, with lower values, ranging from 58 to 66% inhibition, did not differ statistically.

The inhibitory activity against *S*. *delphinii* COUFPI 209 (Fig 1D) was highest in *Trichoderma* sp. T3 UFPIT06 (61%) and *T*. *koningiopsis* UFPIT16 (62%) and lowest in *T*. *orientale* UFPIT09 (6%) and *T*. *orientale* UFPIT17 (8%), the others ranged from 22 to 56%. In *S*. *delphinii* COUFPI 249 (Fig 1D), the fungi *T*. *orientale* (UFPIT01, UFPIT14 with 67% and 69%, respectively) and *Trichoderma* sp. T3 UFPIT06 (69%) presented the best results, with *T*. *orientale* (UFPIT09, UFPIT12, and UFPIT17 with 3, 2 and 3%, respectively) showing less effectiveness.

Most endophytes reduced the MGRI of the plant pathogen colonies, differing statistically from the control treatments (S1 Fig), except for *S*. *delphinii* COUFPI 249 paired with *T*. *orientale* UFPIT09. All isolates stood out in reducing the MGRI for *C*. *truncatum* (S1A Fig), with the highest indices observed for *Trichoderma* sp. T1 (UFPIT04), *T*. *orientale* (UFPIT12), *Trichoderma* sp. T6 (UFPIT13), and *T*. *koningiopsis* (UFPIT19) strains. Against *L*. *theobromae*, only the isolate *T*. *orientale* (UFPIT12) showed the highest MGRI (S1B Fig).

*T*. *longibrachiatum* (UFPIT02), *T*. *koningiopsis* (UFPIT03 and UFPIT10), *Trichoderma* sp. T2 (UFPIT05), *Trichoderma* sp. T3 (UFPIT06), *T*. *orientale* (UFPIT09, UFPIT14, UFPIT15, and UFPIT17), *Trichoderma* sp. T5 (UFPIT11), and *Trichoderma* sp. T6 (UFPIT13) stood out in reducing the MGRI of *M*. *phaseolina*, while *T*. *koningiopsis* (UFPIT03 and UFPIT16) stood out against *M*. *phaseolina* COUFPI 11 (S1C Fig). The MGRI of *S*. *delphinii* COUFPI 209 was reduced for all isolates, and the highest indices were obtained when paired with *T*. *orientale* (UFPIT09, UFPIT12, and UFPIT17), while for *S*. *delphinii* COUFPI 249, *Trichoderma* sp. T1 (UFPIT04) and *Trichoderma* sp. T6 (UFPIT13) showed the highest MGRI (S1D Fig).

Several studies have shown the efficacy of *Trichoderma* strains against *C*. *truncatum*. The species *T*. *harzianum* and *T*. *asperellum* showed percent inhibition of 75 and 73%, respectively, against this pathogen [36]. *T*. *virens*, *T*. *longibrachiatum*, and *T*. *koningii* also inhibited the growth of *C*. *truncatum* with %inhibition of 54 to 81% [37]. Commercial formulations based on *T*. *viride*, *T*. *harzianum*, and *T*. *hamatum* promoted %inhibition ranging from 67 to 81% [38]. In our work, similar results were obtained against this pathogen for the species *T*. *orientale*, *T*. *longibrachiatum*, *T*. *koningiopsis* and unidentified *Trichoderma* isolates.

The species *T*. *harzianum*, *T*. *asperellum*, *T*. *atroviride*, and *T*. *virens* showed percent inhibition in the range of 29 to 54% against *L*. *theobromae* [39], while *T*. *koningii* and *T*. *viride* reached 75 to 80% [40]. *T*. *pseudokoningii*, *T*. *hamatum*, *T*. *koningii*, and *T*. *reesei* also significantly inhibited pathogen growth by 62 to 90% [41]. These values corroborate the %inhibition observed in our study; however, to our knowledge, there are no reports of studies about the biocontrol potential of *T*. *orientale*, *T*. *koningiopsis* and *T*. *longibrachiatum* against *L*. *theobromae*.

In previous studies, *T*. *longibrachiatum* showed a percent inhibition of 58% against *M*. *phaseolina* [42], while *T*. *koningiopsis* strains ranging from 15 to 70% [43]. Similar results were obtained in our study for these species; however, there are no reports about *T*. *orientale* against *M*. *phaseolina*. Swain et al. (2021) investigated the biocontrol potential of *T*. *erinaceum* and *T*. *hebeiensis* against *S*. *delphiii* and found a percent inhibition of approximately 75%, which is the only study of growth inhibition of this pathogen using *Trichoderma* strains [44]. This is the first report that demonstrates the biocontrol potential of the species *T*. *orientale*, *T*. *koningiopsis and T*. *longibrachiatum* against *S*. *delphiii*.

The high percentage of inhibition may be related to the rapid growth of *Trichoderma* spp., which often completely overlap the colonies of *C*. *truncatum* (S2 Fig), *L*. *theobromae* (S3 Fig), and *M*. *phaseolina* COUFPI 10 (S4 Fig) and COUFPI 11 (S5 Fig). The inhibition may also be related to the efficacy of *Trichoderma* spp. in competing for space and nutrients and in parasitizing pathogens [45]. *S*. *delphinii* COUFPI 209 (S6 Fig) and COUFPI 249 (S7 Sig) were very aggressive when competing with *Trichoderma* spp. by space and nutrients; in some cases, they even grew on endophyte colonies. Antibiosis is also an action mechanism present in endophytic fungi of the *Trichoderma* genus, which produce several secondary metabolites with antimicrobial activity used to inhibit the development of plant pathogens [46]. Thus, this action mechanism may also be occurring, justifying the high %inhibition achieved by *Trichoderma* spp.

Several studies have demonstrated the ability of *Trichoderma* strains to inhibit the growth of plant pathogens through antibiosis mechanism [46, 47]. Among the secondary metabolites of *Trichoderma* with antimicrobial activity are syringaresinol [48], HT-2 toxin [49], trigonelline [50], *trans*-zeatin [51], koninginin A [52], koninginin D [53], koninginin E [52], 6-pentyl-α-pyrone [10], gliotoxin, gliovirin, crisopanol, pyrone, 6-pentyl-2H-pyran-2-one, harzianic acid, koningic acid [53], alamethicin, and dermadin [53].

To investigate the antibiosis mechanism performed by *Trichoderma* spp. isolates, we also evaluated whether *Trichoderma* spp. methanolic extracts had inhibitory activity against one of the plant pathogens. For this purpose, we selected the fungus *C*. *truncatum*, the causal agent of anthracnose in soybeans, which is economically relevant. As a result, antifungal activity increased with increasing concentrations of the methanolic extracts, potentiating the %inhibition of the pathogen (S8 Fig). At concentrations of 0.5, 1.0, and 2.0 mg mL^-1^, there was an increase in the %inhibition of *C*. *truncatum* when compared to the dose of 0.0 mg mL^-1^ for all isolates. However, no significant difference from the concentration of 0.5 mg mL^-1^ was observed for four of the isolates (*T*. *longibrachiatum* (UFPIT02), *Trichoderma* sp. T3 (UFPIT06), *Trichoderma* sp. T4 (UFPIT08), and *T*. *orientale* (UFPIT17)).

The extracts of *T*. *koningiopsis* (UFPIT10) and *Trichoderma* sp. T5 (UFPIT11) showed the highest % inhibition, differing statistically from the other isolates at a concentration of 2 mg mL^-1^ (S8 Fig). Interestingly, *T*. *koningiopsis* (UFPIT10) and *Trichoderma* sp. T5 (UFPIT11) did not show the highest activities in the pairing co-culture bioassay (Fig 1A), although both presented %inhibition higher than 50%. The divergence of results may be explained by the variation between the performance of *Trichoderma* isolates of the same species in *in vitro* and *in vivo* bioassays, since the biological control mechanisms of fungi can occur simultaneously, affecting their action [21, 22].

Correlation analysis between co-culture and crude extract bioassays indicated that *Trichoderma* spp. (UFPIT05, UFPIT08, UFPIT11, and UFPIT13), *T*. *koningiopsis* (UFPIT07, UFPIT10, UFPIT16, and UFPIT19), *T*. *orientale* (UFPIT12 and UFPIT15), and *T*. *longibrachiatum* (UFPIT02) had a positive linear relationship, with emphasis on *Trichoderma* sp. T4 (UFPIT08) and *T*. *longibrachiatum* (UFPIT02), which presented r values of 0.97 and 0.96, respectively (S3 Table). On the other hand, *Trichoderma* sp. T1 (UFPIT04), *Trichoderma* sp. T3 (UFPIT06), *T*. *koningiopsis* (UFPIT03), and *T*. *orientale* (UFPIT01, UFPIT09, UFPIT14, UFPIT17, and UFPIT18) showed negative correlations, with emphasis on *Trichoderma* sp. T3 UFPIT06 (r = -1.00), indicating that other biocontrol mechanisms prevailed in relation to antibiosis.

### Untargeted metabolomic analysis revealed antimicrobial metabolites of *Trichoderma* strains from forest species

**Fig 2:**
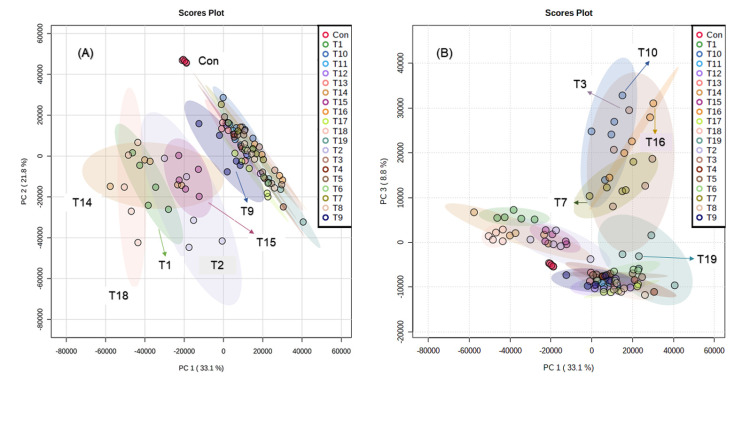
PC1 x PC2 (A) and PC1 x PC3 (B) score plots of metabolic fingerprints of *Trichoderma* spp. cultures generated using MetaboAnalyst, where Con = Control, UFPIT01 = T1, UFPIT02 = T2, UFPIT03 = T3, UFPIT04 = T4, UFPIT05 = T5, UFPIT06 = T6, UFPIT07 = T7, UFPIT08 = T8, UFPIT09 = T9, UFPIT10 = T10, UFPIT11 = T11, UFPIT12 = T12, UFPIT13 = T13, UFPIT14 = T14, UFPIT15 = T15, UFPIT16 = T16, UFPIT17 = T17, UFPIT18 = T18, and UFPIT19 = T19.

The metabolic content of the methanolic extracts of all *Trichoderma* spp. isolates (S8 Fig) were explored by PCA to correlate with the efficiency of inhibition. The PCA showed that 54.9% of the total variation in the data were represented by the first two principal components (Fig 2A). Although a great overlap of the species was observed, samples of *Trichoderma* spp. isolates were clearly separated from the control samples. Clustering of species was performed according to the similarity of their metabolomic profiles and resulted in two large clusters. In the first, with negative scores for PC1, *T*. *longibrachiatum* UFPIT02 (T2) and *T*. *orientale* UFPIT01 (T1), UFPIT14 (T14), and UFPIT18 (T18) overlapped, and *T*. *orientale* UFPIT15 (T15) remained close to this group, overlapping with *T*. *longibrachiatum* UFPIT02 (T2) and *T*. *orientale* UFPIT14 (T14).

A second clustering, with majority positive scores for PC1, was formed by the species *T*. *orientale* (UFPIT12 and UFPIT17) and *T*. *koningiopsi* (UFPIT03, UFPIT07, UFPIT10, UFPIT16, and UFPIT19) and unidentified isolates *Trichoderma* sp. T1 (UFPIT04), *Trichoderma* sp. T2 (UFPIT05), *Trichoderma* sp. T3 (UFPIT06), *Trichoderma* sp. T4 (UFPIT08), *Trichoderma* sp. T5 (UFPIT11), and *Trichoderma* sp. T6 (UFPIT13). *T*. *orientale* UFPIT09 (T9), located near the zero value of PC1, remained intermediate between these two large clusters of species. The PC1 x PC3 score plot (Fig 2B) revealed some clusters similar to those observed in the PC1 x PC2 score plot; however, a new group stood out, with positive scores for PC1, formed by *T*. *koningiopsis* UFPIT03 (T3), UFPIT07 (T7), UFPIT10 (T10), and UFPIT16 (T16), partially overlapping with *T*. *koningiopsis* UFPIT19 (T19).

Altogether, PCA showed that *Trichoderma* spp. from the same species can produce different secondary metabolites, and isolates from different species can produce similar molecules. In the loading plot, the metabolites produced by *Trichoderma* spp. isolates are displayed, and the most distant points represent the metabolites that most influenced the clustering. Molecular signatures of *Trichoderma* spp. isolates were identified according to the elution order, MS/MS fragmentation pattern, molecular formula, and database search. A total of 16 molecules were identified (Table 1 and S11 Fig).

**Table 1:** Secondary metabolites identified in *Trichoderma* strains using UPLC-ESI-Q-TOF-MS.

No.	*m/z*	Retention time (min)	Adduct	MS/MS Fragment masses	Molecular formula	Exact mass	Putative identification	Δ *m/z* (ppm)	Strains	Biological activity	References
1	264.1086	0.68	[M+H-H_2_O]^+^	69.0328, 84.0443, 139.0881, 150.9268	C_11_H_15_N_5_O_4_	281.1119	2-O-Methyladenosine	1.78	All	Anti-inflammatory	[54]
2	118.0863	0.86	[M+H]^+^	59.0705, 60.0838, 99.0061	C_5_H_11_NO_2_	117.0790	Glycine-Betaine	0	All	Plant growth promoter	[55]
3	138.0553	0.91	[M+H]^+^	65.0380, 78.0338, 92.0496, 93.0572, 138.0562	C_7_H_7_NO_2_	137.0480	Trigonelline	-2,19	UFPIT02-UFPIT05, UFPIT07-UFPIT11, and UFPIT13-UFPIT19	Plant growth promoter, antibacterial	[56, 57]
4	220.1196	4.63	[M+H]^+^	97.0356	C_10_H_13_N_5_O	219.1123	*Trans*-Zeatin	-1.37	UFPIT06, UFPIT07, UFPIT10, and UFPIT12	Plant growth promoter, antibacterial, antifungal	[51, 58–60]
5	237.1126	6.80	[M+H-H_2_O]^+^	215.0709, 235.1137, 249.0980	C_13_H_18_O_5_	254.1159	Phomalone	-1.96	All	Antibacterial, antifungal, cytotoxic	[61]
6	247.0957	10.34	[M+H]^+^	56.9367, 162.0252, 166.0648	C_14_H_14_O_4_	246.0895	Columbianetin	-1.22	UFPIT01, UFPIT02, UFPIT09, UFPIT14, UFPIT15, and UFPIT18	Antibacterial, antifungal	[62–65]
7	265.1423	10.55	[M+H]^+^	173.0774, 189.0489, 195.0886, 245.0917	C_15_H_20_O_4_	264.1352	Abscisic acid	3,78	UFPIT04, and UFPIT13	Plant growth promoter, antioxidant, antibacterial, antifungal	[66–68]
8	419.1713	10.83	[M+H]^+^	186.0949, 204.1036, 441.1527	C_22_H_26_O_8_	418.1640	Syringaresinol	-2.87	UFPIT01, UFPIT02, UFPIT13, UFPIT14, UFPIT15, and UFPIT18	Antibacterial, antifungal, anti-inflammatory	[48, 69]
9	281.1754	12.28	[M+H]^+^	123.0814, 133.0655, 160.0524, 175.0431, 177.0254, 245.1556, 263.1660	C_16_H_24_O_4_	280.1688	Brefeldin-A	-4.64	UFPIT03, UFPIT07, UFPIT10, UFPIT16, and UFPIT19	Antiviral, antifungal, antitumoral	[70, 71]
10	305.1721	13.93	[M+Na]^+^	147.0131, 153.0918, 161.0294, 225.0098, 255.1513, 259.1615, 276.1381	C_16_H_26_O_4_	282.1837	Koninginin E	-2.13	UFPIT03, UFPIT07, UFPIT10, UFPIT16, and UFPIT19	Plant growth promoter, antifungal	[52]
11	307.1882	14.11	[M+Na]^+^	133.0656, 267.1297, 289.1688	C_16_H_28_O_4_	284.1993	Koninginin A	-1.76	UFPIT03, UFPIT07, UFPIT10, UFPIT16, and UFPIT19	Plant growth promoter, Antifungal	[52, 72]
12	453.1914	14.23	[M+H-H_2_O]^+^	147.0929, 154.0670, 174.1137, 212.2395, 225.0931, 263.1654, 281.1769, 328.2860, 373.2012, 374.2914, 413.1963, 431.2048	C_26_H_30_O_8_	470.1947	Physodic acid	-1.28	All	Antibacterial	[73]
13	447.2001	14.43	[M+Na]^+^	215.0365, 233.0460, 263.0544, 285.1583, 303.1583, 429.1891, 447.1993	C_22_H_32_O_8_	424.2110	HT-2 Toxin	-3.07	UFPIT07, and UFPIT10	Mycotoxin	[74]
14	318.3013	16.01	[M+H]^+^	97.9455, 150.0253, 264.2691, 282.2798, 286.2755, 294.2803	C_18_H_39_NO_3_	317.2940	Phytosphingosine	-3.15	UFPIT01, UFPIT02, UFPIT09, and UFPIT17	Anti-inflammatory, antibacterial	[75–77]
15	163.0393	16.54	[M+H]^+^	120.9752, 121.0289, 135.0442, 163.0393	C_9_H_6_O_3_	162.0321	4-Hydroxycoumarin	-2.47	All	Antifungal, antibacterial, antioxidant, antitumoral	[78, 79]
16	338.3413	20.72	[M+H]^+^	97.1011, 100.0764, 109.1018, 111.0814, 114.0919, 121.1023, 123.0819, 125.0974, 128.1070, 135.1178, 139.0942	C_8_H_4_O_3_	337.3341	Erucamide	1.19	All	Antibacterial	[80]

The metabolite of *m/z* 264.10862 was found in all *Trichoderma* spp. isolates and identified as 2-O-methyladenosine (Table 1, metabolite 1), a member of the adenosine class that has been isolated from the mycelium of *Cordyceps sinensis* [54]. The metabolite of *m/z* 118.0863 [M+H]^+^ was detected in all *Trichoderma* spp. isolates and identified as glycine-betaine (Table 1, metabolite 2), presenting in the MS/MS spectrum the fragment of *m/z* 59.0705 corresponding to (CH_3_)_3_N^+^ [55]. Betaines are naturally occurring metabolites fundamental to the mitigation of osmotic stress in plants and macro- and microorganisms [55]. The metabolite of *m/z* 138.0553 [M+H]^+^ was identified as trigonelline (Table 1, metabolite 3) and was detected in *T*. *longibrachiatum* (UFPIT02), *Trichoderma* sp. T2 (UFPIT05), *T*. *koningiopsis* (UFPIT07), *Trichoderma* sp. T5 (UFPIT11), *Trichoderma* sp. T6 (UFPIT13), and *T*. *koningiopsis* (UFPIT19) isolates. The MS/MS spectrum was characterized by the fragment of *m/z* 92.0496 [^__^HCOOH]^+^, referring to the carboxylic acid group [56, 57]. This alkaloid is widely used in medicine to protect the liver and heart and to treat hypercholesterolemia [81]. and has already been identified in *T*. *asperellum* fermentation cultures [50].

The metabolite of *m/z* 220.1196 was identified as *trans*-zeatin (Table 1, metabolite 4), and it was detected in *Trichoderma* sp. T3 (UFPIT06), *T*. *koningiopsis* (UFPIT07 and UFPIT10), and *T*. *orientale* (UFPIT12) isolates. This cytokinin, previously reported in *Trichoderma* strains, can be used for plant growth stimulation and affects host plant phytohormones to enhance plant resistance against pathogens [50, 58]. The metabolite of *m/z* 237.1126 [M+H]^+^, identified as phomalone (Table 1, metabolite 5) and detected in all *Trichoderma* spp., is a common metabolite in many fungal species, with anti-inflammatory, antibacterial, antifungal, and antialgal activities [61]. The metabolite of *m/z* 247.0957 detected in *T*. *orientale* (UFPIT01), *T*. *longibrachiatum* (UFPIT02), and *T*. *orientale* (UFPIT09, UFPIT14, UFPIT15, and UFPIT18) isolates was identified as columbianetin (Table 1, metabolite 6), a phytoalexin with diverse biological activities [61]. and that has been extracted from cultures of an endophytic strain of *Annulohypoxylon ilanense* [48].

The metabolite of *m/z* 265.1423 was identified as abscisic acid (Table 1, metabolite 7), a phytohormone directly involved in plant-microorganism interactions, improving the defense system and plant development [66], and it was detected only in *Trichoderma* sp. T1 (UFPIT04) and *Trichoderma* sp. T6 (UFPIT13) isolates. The metabolite of *m/z* 419.1713 detected in *T*. *orientale* (UFPIT01), *T*. *longibrachiatum* (UFPIT02), *Trichoderma* sp. T6 (UFPIT13), and *T*. *orientale* (UFPIT14, UFPIT15, and UFPIT18) isolates were identified as syringaresinol (Table 1, metabolite 8), a lignan that has been found to be a secondary metabolite of an endophytic strain of *A*. *ilanense* [48]. The metabolite of *m/z* 281.1754, present in *T*. *koningiopsis* (UFPIT03, UFPIT07, UFPIT10, UFPIT16, and UFPIT19), was identified as brefeldin-A (BFA) (Table 1, metabolite 9) and showed MS/MS spectrum with fragments of *m/z* 263.1660 [M + H-H_2_O]^+^ and 245.1556 [M + H - 2H_2_O]^+^ formed by the BFA dehydration pathway [70]. This metabolite is an antibiotic already isolated in several fungal genera, such as *Alternaria*, *Ascochyta*, *Penicillium*, *Curvularia*, *Cercospora*, and *Phyllosticta*. BFA has been reported to have important bioactivities, such as antibiotics, antivirals, cytostatics, antimitotics and antitumors [70].

The metabolites of *m/z* 305.1721 and *m/z* 307.1882 were identified as koninginin E and koninginin A, respectively (Table 1, metabolites 10 and 11). Koninginins are secondary metabolites belonging to the group of polyketides that are bioactive against several plant pathogens. Koninginin E has already exhibited activity against *Gaeumannomyces graminis* var. tritici, while koninginin A already exhibited activity against *G*. *graminis* var. tritici, *F*. *oxysporum*, *F*. *solani* and *Alternaria panax* [52]. Koninginins A and E were detected in *T*. *koningiopsis* (UFPIT03, UFPIT07, UFPIT10, UFPIT16, and UFPIT19 isolates). The metabolite of *m/z* 453.1914 was identified as physodic acid (Table 1, metabolite 12) and was detected in all *Trichoderma* spp. Physodic acid is a metabolite belonging to the depsidone group, and its antibacterial activity against *S*. *aureus* has been previously reported [73]. The metabolite of *m/z* 447.2001, detected in *T*. *koningiopsis* (UFPIT07 and UFPIT10), was identified as HT-2 toxin (Table 1, metabolite 13). The MS/MS spectrum was characterized by fragments of *m/z* 215.0365 [HT2—isoval acid—acetic acid—H_2_O - CH_2_O + H]^+^, 233.0460 [C_14_H_16_O_3_ + H]^+^ and 263.0544 [HT2—isoval acid—acetic acid + H]^+^ [74]. HT-2 toxin is a secondary metabolite found mainly in fungi of the *Fusarium* genus and is classified as a trichothecene type A mycotoxin [74].

The metabolite of *m/z* 318.3013 identified as phytosphingosine (Table 1, metabolite 14) presented MS/MS spectrum with fragments of *m/z* 264.2691 [M+H-DHO-2H_2_O]^+^ and 282.2798 [M+H-DHO-H_2_O]^+^, formed by the phytosphingosine dehydration pathway [75]. Phytosphingosine is a long-chain sphingolipid present in microorganisms, plants, and some mammalian tissues with antimicrobial and anti-inflammatory activity [76] and was produced by *T*. *orientale* (UFPIT01, UFPIT09, and UFPIT17) and *T*. *longibrachiatum* (UFPIT02) isolates. The metabolite of *m/z* 163.0393 detected in all *Trichoderma* spp. was identified as 4-hydroxycoumarin (Table 1, metabolite 15), which is a fungal metabolite obtained from the precursor coumarin [79] that has important biological activities [78]. The metabolite of *m/z* 338.3413 detected in all *Trichoderma* spp. was identified as erucamide (Table 1, metabolite 16) and has been reported in *T*. *longibrachiatum* [80].

Altogether, the plethora of and the variety of secondary metabolites identified in the present study highlight how *Trichoderma* strains are capable of producing metabolites with different biological activities, which makes them very promising not only for the biocontrol of plant diseases but also for their application in medical, pharmaceutical and industrial biotechnology. Forest species from the Cerrado-Caatinga ecotone are rich in genetic resources and have diverse fauna and flora, with enormous biotechnological potential, including their diversity of endophytic fungi [26].

## Conclusions

*Trichoderma* strains from the Cerrado-Caatinga ecotone revealed significant biocontrol potential against crop pathogenic fungi through antibiosis and multiple mechanisms, with possibilities of being used in formulations of biological products for the treatment of plant diseases. Metabolomic analysis proved to be effective in differentiating *Trichoderma* strains, in addition to identifying a variety of secondary metabolites with antimicrobial activity and other different bioactivities, demonstrating the importance of studying the biological resources of this area, which are still underexplored. Additionally, new bioactive metabolites can still be discovered, since this mutualistic association of endophytic fungi with their hosts is controlled by the genes of both organisms and modulated by the environment in which they live.

## Supporting information

S1 Fig Mycelial growth rate index (MGRI) of *C*. *truncatum* (A), *L*. *theobromae* (B), *M*. *phaseolina* COUFPI 10 and COUFPI 11 (C), and *S*. *delphinii* COUFPI 209 and COUFPI 249 (D) paired with *Trichoderma* strains. Means followed by the same letter do not differ from each other by the Scott–Knott test at the 5% probability level. The coefficients of variation (CVs) were 20.51% for *L*. *theobromae*, 15.99% for *M*. *phaseolina* COUFPI 10, 19.13% for *M*. *phaseolina* COUFPI 11, 10.68% for *S*. *delphinii* COUFPI 209, and 9.54% for *S*. *delphinii* COUFPI 249. Different lowercase letters indicate a significant difference between *Trichoderma* spp. Different capital letters indicate a significant difference between *Trichoderma* spp.
(TIF)

S2 Fig *In vitro* antagonism bioassays of *Trichoderma* strains against *C*. *truncatum*. (TIF)

S3 Fig *In vitro* antagonism bioassays of *Trichoderma* strains against *L*. *theobromae*. (TIF)

S4 Fig *In vitro* antagonism bioassays of *Trichoderma* strains against *M*. *phaseolina* COUFPI 10. (TIF)

S5 Fig *In vitro* antagonism bioassays of *Trichoderma* strains against *M*. *phaseolina* COUFPI 11. (TIF)

S6 Fig *In vitro* antagonism bioassays of *Trichoderma* spp. against *S*. *delphinii* COUFPI 209. (TIF)

S7 Fig *In vitro* antagonism bioassays of *Trichoderma* spp. against *S*. *delphinii* COUFPI 249. (TIF)

S8 Fig Regression graph based on % inhibition of organic extracts of *Trichoderma* spp. against *C*. *truncatum*. The coefficients of variation (CVs) were 4.65% for the concentration of 0.5 mg mL^-1^, 8.45% for 1.0 mg mL^-1^ and 9.36% for 2.0 mg mL^-1^.
(TIF)

S9 Fig PC1 x PC2 (A) and PC1 x PC3 (B) loading plots of metabolic fingerprints of *Trichoderma* spp. cultures generated using MetaboAnalyst. Con = Control, UFPIT01 = T1, UFPIT02 = T2, UFPIT03 = T3, UFPIT04 = T4, UFPIT05 = T5, UFPIT06 = T6, UFPIT07 = T7, UFPIT08 = T8, UFPIT09 = T9, UFPIT10 = T10, UFPIT11 = T11, UFPIT12 = T12, UFPIT13 = T13, UFPIT14 = T14, UFPIT15 = T15, UFPIT16 = T16, UFPIT17 = T17, UFPIT18 = T18, and UFPIT19 = T19.
(TIF)

S10 Fig UPLC-ESI-Q-TOF-MS chromatograms of *Trichoderma* spp. isolates. (TIF)

S11 Fig Structure of secondary metabolites identified in *Trichoderma* spp. using UPLC-ESI-Q-TOF-MS. (TIF)

S1 Table *Trichoderma* spp. isolates used in this study [29]. (DOCX)

S2 Table *Trichoderma* spp. isolated from leaves of forest species in an area of the Cerrado-Caatinga ecotone [26]. (DOCX)

S3 Table Pearson’s linear correlations between co-culture and crude extract bioassays based on percent inhibition of *Trichoderma* strains against *C*. *truncatum*. (DOCX)

## References

[1] Ghorbanpour M, Omidvari M, Abbaszadeh-Dahaji P, Omidvar R, Kariman K. Mechanisms underlying the protective effects of beneficial fungi against plant diseases. Biol Control. 2018; 117:147–157. doi: 10.1016/j.biocontrol.2017.11.006

[2] Villa F, Cappitelli F, Cortesi P, Kunova A. Fungal biofilms: Targets for the development of novel strategies in plant disease management. Front Microbiol. 2017; 8:654. doi: 10.3389/fmicb.2017.00654 28450858 PMC5390024

[3] Nagy K, Zheng C, Bolognesi C, Ádám B. Interlaboratory evaluation of the genotoxic properties of pencycuron, a commonly used phenylurea fungicide. Sci Total Environ. 2019; 647:1052–1057. doi: 10.1016/j.scitotenv.2018.08.067 30180313

[4] Das S, Pattanayak S. Integrated disease management on grapes–a pioneer of a reformed movement toward sustainability. Int J Curr Microbiol Appl Sci. 2020; 9:993–1005. doi: 10.20546/ijcmas.2020.905.109

[5] Salim HA, Simon S, Lal AA, Abdulrahman AL. Effectiveness of some integrated disease management factors (IDM) on *Fusarium* wilt infected tomato. J Sci Agri. 2017; 1:244–248. doi: 10.25081/jsa.2017.v1.820

[6] Legrand F, Picot A, Cobo-Díaz JF, Chen W, Le Floch G. Challenges facing the biological control strategies for the management of *Fusarium* Head Blight of cereals caused by *F*. *graminearum*. Biol Control. 2017; 113:26–38. doi: 10.1016/j.biocontrol.2017.06.011

[7] Leiter É, Gáll T, Csernoch L, Pócsi I. Biofungicide utilizations of antifungal proteins of filamentous ascomycetes: current and foreseeable future developments. Biocontrol. 2017; 62:125–138. doi: 10.1007/s10526-016-9781-9

[8] Kyekyeku JO, Kusari S, Adosraku RK, Bullach A, Golz C, Strohmann C, et al. Antibacterial secondary metabolites from an endophytic fungus, *Fusarium solani* JK10. Fitoterapia. 2017; 119:108–114. doi: 10.1016/j.fitote.2017.04.007 28392268

[9] Yao YQ, Lan F, Qiao YM, Wei JG, Huang RS, Li LB. Endophytic fungi harbored in the root of *Sophora tonkinensis* Gapnep: diversity and biocontrol potential against phytopathogens. Microbiologyopen. 2017; 6:437. doi: 10.1002/mbo3.437 28299913 PMC5458465

[10] Huang X, He J, Yan X, Hong Q, Chen K, He Q, et al. Microbial catabolism of chemical herbicides: microbial resources, metabolic pathways and catabolic genes. Pestic Biochem Physiol. 2017; 143:272–297. doi: 10.1016/j.pestbp.2016.11.010 29183604

[11] Macías-Rubalcava ML, Sánchez-Fernández RE. Secondary metabolites of endophytic *Xylaria* species with potential applications in medicine and agriculture. World J Microbiol Biotechnol. 2017; 33:15. doi: 10.1007/s11274-016-2174-5 27896581

[12] Harrison JG & Griffin EA. The diversity and distribution of endophytes across biomes, plant phylogeny and host tissues: how far have we come and where do we go from here? Environmental microbiology. 2020; 22:2107–2123. doi: 10.1111/1462-2920.14968 32115818 PMC7679042

[13] Noriler SA, Savi DC, Aluizio R, Palácio-Cortes AM, Possiede YM, Glienke C. Bioprospecting and structure of fungal endophyte communities found in the Brazilian biomes, Pantanal, and Cerrado. Frontiers in microbiology. 2018; 9: 1526. doi: 10.3389/fmicb.2018.01526 30087658 PMC6066559

[14] Zonas de transição [cited 19 December 2021]. In: World Wide Fund for Nature (WWF) [Internet]. Available from: https://www.wwf.org.br/natureza_brasileira/questoes_ambientais/biomas/bioma_transicao.

[15] Kark S. Effects of Ecotones on Biodiversity. In: Reference module in life sciences. Oxford, England, Science Direct. 2017; 1–7.

[16] Myers N, Mittermeier RA, Mittermeier CG, Fonseca GA, Kent J. Biodiversity hotspots for conservation priorities. Nature. 2000; 403:853–858. doi: 10.1038/35002501 10706275

[17] Biodiversidade do cerrado [cited 19 December 2021]. In: CBC ICMBio [Internet]. Available from: https://www.icmbio.gov.br/cbc/conservacao-da-biodiversidade/biodiversidade.html.

[18] Caatinga [cited 19 December 2021]. In: Ministério do Meio Ambiente [Internet]. Available from: https://antigo.mma.gov.br/biomas/caatinga.html.

[19] Lapola DM, Martinelli LA, Peres CA, Ometto JPHB, Ferreira ME, Nobre CA, et al. Pervasive transition of the Brazilian land-use system. Nat Clim Chang. 2013; 4:27–35. doi: 10.1038/NCLIMATE2056

[20] Berg G. Plant–microbe interactions promoting plant growth and health: perspectives for controlled use of microorganisms in agriculture. Applied microbiology and biotechnology. 2009; 84: 11–18. doi: 10.1007/s00253-009-2092-7 19568745

[21] Benítez T, Rincón AM, Limón MC, Codon AC. Biocontrol mechanisms of *Trichoderma* strains. International microbiology. 2004; 7: 249–260. 15666245

[22] Druzhinina IS, Seidl-Seiboth V, Herrera-Estrella A, Horwitz BA, Kenerley CM, Monte E. *Trichoderma*: The genomics of opportunistic success. Nat Rev Microbiol. 2011; 9:749. doi: 10.1038/nrmicro2637 21921934

[23] Inglis PW, Mello SC, Martins I, Silva JB, Macêdo K, Sifuentes DN, et al. *Trichoderma* from Brazilian garlic and onion crop soils and description of two new species: *Trichoderma azevedoi* and *Trichoderma peberdyi*. PloS One. 2020; 15: e0228485. doi: 10.1371/journal.pone.0228485 32130211 PMC7055844

[24] Kredics L, Hatvani L, Naeimi S, Körmöczi P, Manczinger L, Vágvölgyi C, et al. Biodiversity of the genus *Hypocrea/Trichoderma* in different habitats. In: Biotechnology and biology of *Trichoderma*. 2014; 3–24. doi: 10.1016/B978-0-444-59576-8.00001–1

[25] Ma J, Tsegaye E, Li M, Wu B, Jiang X. Biodiversity of *Trichoderma* from grassland and forest ecosystems in Northern Xinjiang, China. 3 Biotech. 2020; 10: 1–13. doi: 10.1007/s13205-019-1978-z 32821644 PMC7392985

[26] Silva HF, Santos AMG, Santos MVOD, Bezerra JL, Luz EDMN. Seasonal variation in the occurrence of fungi associated with forest species in a Cerrado-Caatinga transition area. Revista Árvore. 2020; 44. doi: 10.1590/1806-908820200000009

[27] Silva HF, Costa EM, Santos AMG, Amaral ACT, Oliveira RJV, Bezerra JL, et al. Molecular identification and phylogenetic analysis of *Trichoderma* isolates obtained from woody plants of the semi-arid of Northeast Brazil. Nova Hedwigia. 2021; 112:485–500. doi: 10.1127/nova_hedwigia/2021/0622

[28] Silva HF, Santos AMG, Amaral ACT, Bezerra JL, Luz EDMN. Bioprospection of *Trichoderma* spp. originating from a Cerrado-Caatinga ecotone on *Colletotrichum truncatum*, in soybean. Revista Brasileira de Ciências Agrárias (Agrária). 2020; 15. doi: 10.5039/agraria.v15i1a7680

[29] Mota JM, Melo MP, Silva FFS, Sousa EMJ, Sousa ES, Barguil BM, et al. Fungal diversity in lima bean seeds. Revista Brasileira de Engenharia de Biossistemas. 2017; 11:79–87.

[30] Severo R, Shibutani LJ, Sousa ES, Matos KS, Beserra Júnior JEA, Melo MP. *Sclerotium delphinii* causing concentric leaf spots in *Piper nigrum* in Brazil. Australas Plant Pathol. 2021. doi: 10.1007/s13313-021-00820-1 34608354 PMC8481109

[31] Mariano RLR. Métodos de seleção “*in vitro*” para controle microbiológico. Revisão Anual de Patologia de Plantas, Passo Fundo. 1993; 1:369–409.

[32] Camporota P. Antagonism *in vitro* of *Trichoderma* spp. vis-a-vis *Rhizoctonia solani* Kuhln. Agronomie. 1985; 5:613–620.

[33] Oliveira JA. Efeito do tratamento fungicida em sementes no controle de tombamento de plântulas de pepino (*Cucumis sativa* L.) e pimentão (*Capsicum annanum* L.). M.Sc. Thesis, Federal University of Lavras. 1991. Available from: http://repositorio.ufla.br/handle/1/33483.

[34] Fernandes EG, Pereira OL, Silva CC, Bento CBP, Queiroz MV. Diversity of endophytic fungi in *Glycine max*. Microbiol Res. 2015; 181:84–92. doi: 10.1016/j.micres.2015.05.010 26111593

[35] Tam EW, Chen JH, Lau EC, Ngan AH, Fung KS, Lee KC, et al. Misidentification of *Aspergillus nomius* and *Aspergillus tamarii* as *Aspergillus flavus*: characterization by internal transcribed spacer, β-tubulin, and calmodulin gene sequencing, metabolic fingerprinting, and matrix-assisted laser desorption ionization–time of flight mass spectrometry. J Clin Microbiol. 2014; 52: 1153–1160. doi: 10.1128/JCM.03258-13 24452174 PMC3993464

[36] Yadav M, Dubey MK, Upadhyay RS. Systemic Resistance in Chilli Pepper against Anthracnose (Caused by *Colletotrichum truncatum*) induced by *Trichoderma harzianum*, *Trichoderma asperellum* and *Paenibacillus dendritiformis*. Journal of Fungi. 2021; 7: 307. doi: 10.3390/jof7040307 33923782 PMC8073547

[37] Begum MM, Sariah M, Abidin ZMA, Puteh AB, Rahman MA. Antagonistic potential of selected fungal and bacterial biocontrol agents against *Colletotrichum truncatum* of soybean seeds. Pertanica J. Trop. Agric. Sci. 2008; 31: 45–53.

[38] Jagtap GP, Gavate DS, Dey U. Control of *Colletotrichum truncatum* causing anthracnose/pod blight of soybean by aqueous leaf extracts and biocontrol agents. Legume Research-An International Journal. 2014; 37: 329–334. doi: 10.5958/j.0976-0571.37.3.050

[39] Wanjiku EK, Waceke JW, Mbaka JN. Suppression of Stem-End Rot on Avocado Fruit Using *Trichoderma* spp. in the Central Highlands of Kenya. Advances in Agriculture. 2021; 2021. d doi: 10.1155/2021/8867858

[40] Bhadra M, Khair A, Hossain MA, Sikder MM. Efficacy of *Trichoderma* spp. and fungicides against *Lasiodiplodia theobromae*. Bangladesh Journal of Scientific and Industrial Research. 2014; 49: 125–130. doi: 10.3329/bjsir.v49i2.22008

[41] Thangavelu R, Sangeetha G, Mustaffa MM. Cross-infection potential of crown rot pathogen (*Lasiodiplodia theobromae*) isolates and their management using potential native bioagents in banana. Australasian Plant Pathology. 2007; 36: 595–605.

[42] Sridharan AP, Sugitha T, Karthikeyan G, Nakkeeran S, Sivakumar U. Metabolites of *Trichoderma longibrachiatum* EF5 inhibits soil borne pathogen, *Macrophomina phaseolina* by triggering amino sugar metabolism. Microbial Pathogenesis. 2021; 150: 104714. doi: 10.1016/j.micpath.2020.104714 33383148

[43] Saravanakumar K & Wang M. Isolation and molecular identification of *Trichoderma* species from wetland soil and their antagonistic activity against phytopathogens. Physiological and Molecular Plant Pathology. 2020; 109: 101458. doi: 10.1016/j.pmpp.2020.101458

[44] Swain H, Adak T, Mukherjee AK, Sarangi S, Samal P, Khandual A, et al. Seed biopriming with *Trichoderma* strains isolated from tree bark improves plant growth, antioxidative defense system in rice and enhance straw degradation capacity. Frontiers in Microbiology. 2021; 12: 240. doi: 10.3389/fmicb.2021.633881 33717027 PMC7952651

[45] Kottb M, Gigolashvili T, GRoßkinsky DK, Piechulla B. *Trichoderma* volatiles effecting *Arabidopsis*: from inhibition to protection against phytopathogenic fungi. Front Microbiol. 2015; 6:995. doi: 10.3389/fmicb.2015.00995 26483761 PMC4586454

[46] Mendoza JLH, Pérez MIS, Prieto JMG, Velásquez JDQ, Olivares JGG, Langarica HRG. Antibiosis of *Trichoderma* spp. strains native to northeastern Mexico against the pathogenic fungus *Macrophomina phaseolina*. Braz J Microbiol. 2015; 46:1093–1101. doi: 10.1590/S1517-838246420120177 26691467 PMC4704620

[47] Khaledi N, Taheri P. Biocontrol mechanisms of *Trichoderma harzianum* against soybean charcoal rot caused by *Macrophomina phaseolina*. J Plant Prot Res. 2016; 56:21–31. doi: 10.1515/jppr-2016-0004

[48] Cheng M.J.; Wu M.D.; Chen J.J.; Hsieh S.Y.; Yuan G.F.; Chen I.S.; et al. Secondary metabolites from the endophytic fungus of Annulohypoxylon ilanense. Chem. Nat. Compd. 2013, 49, 523–525. doi: 10.1007/s10600-013-0658-1

[49] Tian Y, Yu D, Liu N, Tang Y, Yan Z, Wu A. Confrontation assays and mycotoxin treatment reveal antagonistic activities of *Trichoderma* and the fate of *Fusarium* mycotoxins in microbial interaction. Environmental Pollution. 2020; 267: 115559. doi: 10.1016/j.envpol.2020.115559 33254604

[50] Vinci G, Cozzolino V, Mazzei P, Monda H, Spaccini R, Piccolo A. An alternative to mineral phosphorus fertilizers: The combined effects of *Trichoderma harzianum* and compost on Zea mays, as revealed by 1H NMR and GC–MS metabolomics. PloS One. 2018; 13: e0209664. doi: 10.1371/journal.pone.0209664 30589863 PMC6307717

[51] Ni M, Wu Q, Wang GS, Liu QQ, Yu MX, Tang J. Analysis of metabolic changes in *Trichoderma asperellum* TJ01 at different fermentation time-points by LC-QQQ-MS. J Environ Sci Health, Part B. 2019; 54:20–26. doi: 10.1080/03601234.2018.1507227 30896331

[52] Khan RAA, Najeeb S, Hussain S, Xie B, Li Y. Bioactive secondary metabolites from *Trichoderma* spp. against phytopathogenic fungi. Microorganisms. 2020; 8:817. doi: 10.3390/microorganisms8060817 32486107 PMC7356054

[53] Reino JL, Guerrero RF, Hernández-Galán R, Collado IG. Secondary metabolites from species of the biocontrol agent *Trichoderma*. Phytochem Rev. 2008; 7:89–123.

[54] Yang ML, Kuo PC, Hwang TL, Wu TS. Anti-inflammatory principles from *Cordyceps sinensis*. Journal of Natural Products. 2011; 74:1996–2000. doi: 10.1021/np100902f 21848266

[55] Wood KV, Bonham CC, Miles D, Rothwell AP, Peel G, Wood BC, et al. Characterization of betaines using electrospray MS/MS. Phytochemistry. 2002; 59:759–765. doi: 10.1016/s0031-9422(02)00049-3 11909633

[56] Liu MH, Tong X, Wang JX, Zou W, Cao H, Su WW. Rapid separation and identification of multiple constituents in traditional Chinese medicine formula Shenqi Fuzheng Injection by ultra-fast liquid chromatography combined with quadrupole-time-of-flight mass spectrometry. J Pharm Biomed Anal. 2013; 74:141–155. doi: 10.1016/j.jpba.2012.10.024 23245245

[57] Lu X, Zheng Y, Wen F, Huang W, Chen X, Ruan S, et al. Study of the active ingredients and mechanism of *Sparganii rhizoma* in gastric cancer based on HPLC-Q-TOF–MS/MS and network pharmacology. Sci Rep. 2021; 11:1–17. doi: 10.1038/s41598-020-79139-8 33479376 PMC7820434

[58] Illescas M, Pedrero-Méndez A, Pitorini-Bovolini M, Hermosa R, Monte E. Phytohormone Production Profiles in *Trichoderma* Species and Their Relationship to Wheat Plant Responses to Water Stress. Pathogens. 2021; 10:991, 2021. doi: 10.3390/pathogens10080991 34451455 PMC8400765

[59] Großkinsky D, Edelsbrunner K, Pfeifhofer H, Van der Graaff E, Roitsch T. *Cis*- and *trans*-zeatin differentially modulate plant immunity. Plant Signaling & Behavior. 2013; 8:e24798. doi: 10.4161/psb.24798 23656869 PMC3906432

[60] Ahmed EA, Hassan EA, El Tobgy KMK, Ramadan EM. Evaluation of rhizobacteria of some medicinal plants for plant growth promotion and biological control. Annals of Agricultural Sciences. 2014; 59:273–280. doi: 10.1016/j.aoas.2014.11.016

[61] Zhong TH, Zeng XM, Feng SB, Zhang HT, Zhang YH, Luo ZH, et al. Three new phomalone derivatives from a deep-sea-derived fungus Alternaria sp. MCCC 3A00467. Nat Prod Res. 2020; 1–5. doi: 10.1080/14786419.2020.1771706 32524853

[62] Zhang L, Ge Y, Li J, Hao J, Wang H, He J, et al. Simultaneous determination of columbianetin-β-d-glucopyranoside and columbianetin in a biological sample by high-performance liquid chromatography with fluorescence detection and identification of other columbianetin-β-d-glucopyranoside metabolites by ultra high-performance liquid chromatography coupled with quadrupole-time of flight mass spectrometry. J Pharm Biomed Anal. 2018; 153:221–231. doi: 10.1016/j.jpba.2018.02.055 29506005

[63] Nicoletti R, Fiorentino A. Plant bioactive metabolites and drugs produced by endophytic fungi of *Spermatophyta*. Agriculture. 2015; 5:918–970.

[64] Zhu JJ & Jiang JG. Pharmacological and nutritional effects of natural coumarins and their structure–activity relationships. Molecular nutrition & food research. 2018; 62:1701073. doi: 10.1002/mnfr.201701073 29750855

[65] Souza SM, Delle Monache F, Smânia A. Antibacterial activity of coumarins. Zeitschrift fuer Naturforschung C. 2005; 60:693–700. doi: 10.1515/znc-2005-9-1006 16320610

[66] Guzmán-Guzmán P, Porras-Troncoso MD, Olmedo-Monfil V, Herrera-Estrella A. *Trichoderma* species: versatile plant symbionts. J Phytopathol. 2019; 109:6–16. doi: 10.1094/PHYTO-07-18-0218-RVW 30412012

[67] Thiruvengadam M, Baskar V, Kim SH, Chung IM. Effects of abscisic acid, jasmonic acid and salicylic acid on the content of phytochemicals and their gene expression profiles and biological activity in turnip (*Brassica rapa* ssp. rapa). Plant Growth Regulation. 2016; 80:377–390. doi: 10.1007/s10725-016-0178-7

[68] Phukhamsakda C, Macabeo APG, Yuyama KT, Hyde KD, Stadler M. Biofilm inhibitory abscisic acid derivatives from the plant-associated Dothideomycete fungus, *Roussoella* sp. Molecules. 2018; 23:2190. doi: 10.3390/molecules23092190 30200229 PMC6225182

[69] Oh JH, Joo YH, Karadeniz F, Ko J, Kong CS. Syringaresinol inhibits UVA-induced MMP-1 expression by suppression of MAPK/AP-1 signaling in HaCaT keratinocytes and human dermal fibroblasts. International journal of molecular sciences. 2020; 21:3981. doi: 10.3390/ijms21113981 32492931 PMC7312901

[70] Fang M, Wang J, Huang Y, Zhao Y. Rapid Screening and Identification of Brefeldin A in Endophytic Fungi Using HPLC–MS/MS. Frontiers of Chemistry in China. 2006; 1:15–19.

[71] Anadu NO, Davisson VJ, Cushman M. Synthesis and anticancer activity of brefeldin A ester derivatives. Journal of medicinal chemistry. 2006; 49: 3897–3905. doi: 10.1021/jm0602817 16789745

[72] Li T, Tang J, Karuppiah V, Li Y, Xu N, Chen J. Co-culture of *Trichoderma atroviride* SG3403 and *Bacillus subtilis* 22 improves the production of antifungal secondary metabolites. Biological Control. 2020; 140: 104122. doi: 10.1016/j.biocontrol.2019.104122

[73] Dieu A, Mambu L, Champavier Y, Chaleix V, Sol V, Gloaguen V, et al. Antibacterial activity of the lichens *Usnea florida* and *Flavoparmelia caperata* (Parmeliaceae). Nat Prod Res. 2020; 34:3358–3362. doi: 10.1080/14786419.2018.1561678 30676068

[74] Meng-Reiterer J, Varga E, Nathanail AV, Bueschl C, Rechthaler J, McCormick SP, et al. Tracing the metabolism of HT-2 toxin and T-2 toxin in barley by isotope-assisted untargeted screening and quantitative LC-HRMS analysis. Anal Bioanal Chem. 2015; 407:8019–8033. doi: 10.1007/s00216-015-8975-9 26335000 PMC4595538

[75] Dapic I, Brkljacic L, Jakasa I, Kobetic R. Characterization of ceramides with phytosphingosine backbone by hydrogen-deuterium exchange mass spectrometry. Croat Chem Acta. 2019; 92:1E–1E. doi: 10.5562/cca3506

[76] Kondo N, Ohno Y, Yamagata M, Obara T, Seki N, Kitamura T, et al. Identification of the phytosphingosine metabolic pathway leading to odd-numbered fatty acids. Nat Commun. 2014; 5:1–13. doi: 10.1038/ncomms6338 25345524

[77] Choi HK, Cho YH, Lee EO, Kim JW, Park CS. Phytosphingosine enhances moisture level in human skin barrier through stimulation of the filaggrin biosynthesis and degradation leading to NMF formation. Archives of dermatological research. 2017; 309: 795–803. doi: 10.1007/s00403-017-1782-8 28936777

[78] Kaur J, Famta P, Khurana N, Vyas M, Khatik GL. Biomedical applications of 4-hydroxycoumarin as a fungal metabolite and its derivatives. New Fut Devel Microbiol Biotech Bioeng. Elsevier. 2020; 209–218. doi: 10.1016/B978-0-12-821006-2.00016–9

[79] Obaiah N, Bodke YD, Telkar S. Synthesis of 3‐[(1H‐Benzimidazol‐2‐ylsulfanyl)(aryl) methyl]‐4‐hydroxycoumarin derivatives as potent bioactive molecules. ChemistrySelect. 2020; 5:178–184. doi: 10.1002/slct.201903472

[80] Xie Y, Peng Q, Ji Y, Xie A, Yang L, Mu S, et al. Isolation and identification of antibacterial bioactive compounds from *Bacillus megaterium* L2. Front Microbiol. 2021; 12. doi: 10.3389/fmicb.2021.645484 33841370 PMC8024468

[81] Farid MM, Yang X, Kuboyama T, Tohda C. Trigonelline recovers memory function in Alzheimer’s disease model mice: evidence of brain penetration and target molecule. Sci Rep. 2020; 10:1–10. doi: 10.1038/s41598-019-56847-4 33009465 PMC7532147

