# Supplementary figures and images for: Endophytic Trichoderma strains isolated from forest species of the Cerrado-Caatinga ecotone are potential biocontrol agents against crop pathogenic fungi
Source: PLoS One. 2022 Apr 15;17(4):e0265824. doi: 10.1371/journal.pone.0265824 (PMC9012399; doi:10.1371/journal.pone.0265824)

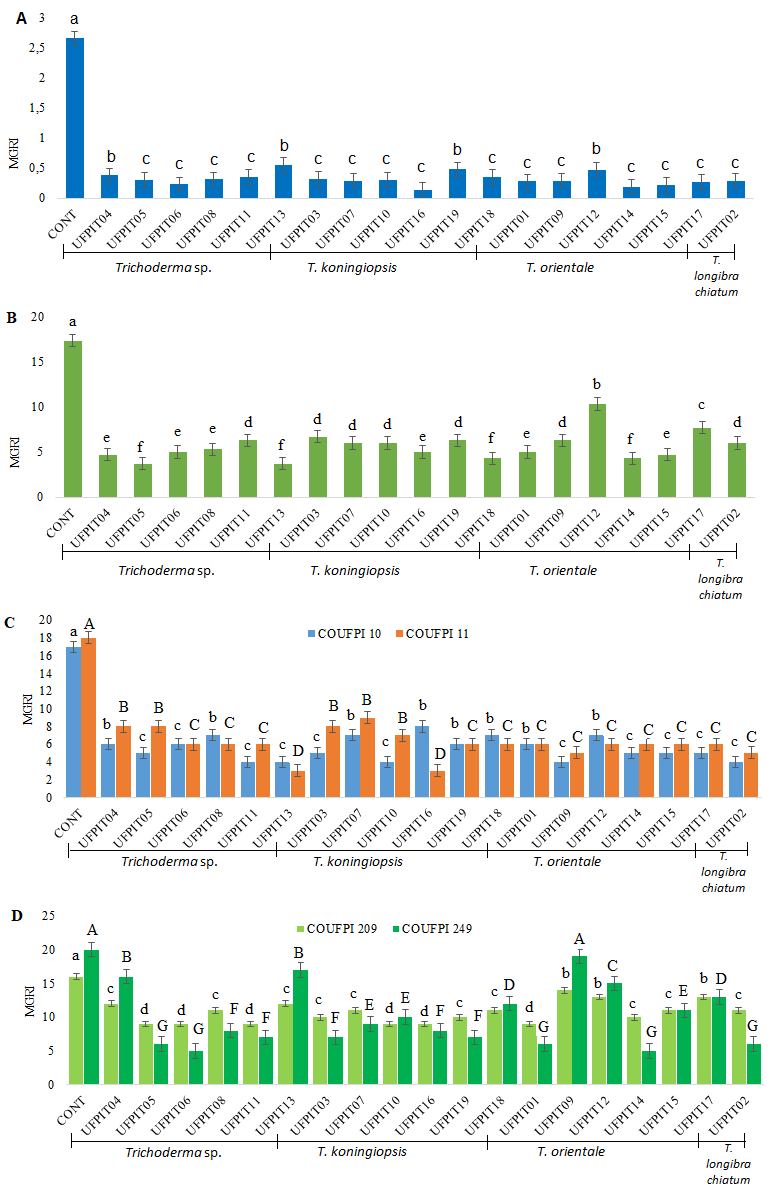

Supplement: S1 Fig — Mycelial growth rate index (MGRI) of C. truncatum (A), L. theobromae (B), M. phaseolina COUFPI 10 and COUFPI 11 (C), and S. delphinii COUFPI 209 and COUFPI 249 (D) paired with Trichoderma strains. Means followed by the same letter do not differ from each other by the Scott–Knott test at the 5% probability level. The coefficients of variation (CVs) were 20.51% for L. theobromae, 15.99% for M. phaseolina COUFPI 10, 19.13% for M. phaseolina COUFPI 11, 10.68% for S. delphinii COUFPI 209, and 9.54% for S. delphinii COUFPI 249. Different lowercase letters indicate a significant difference between Trichoderma spp. Different capital letters indicate a significant difference between Trichoderma spp. (TIF) [file pone.0265824.s001.tif]

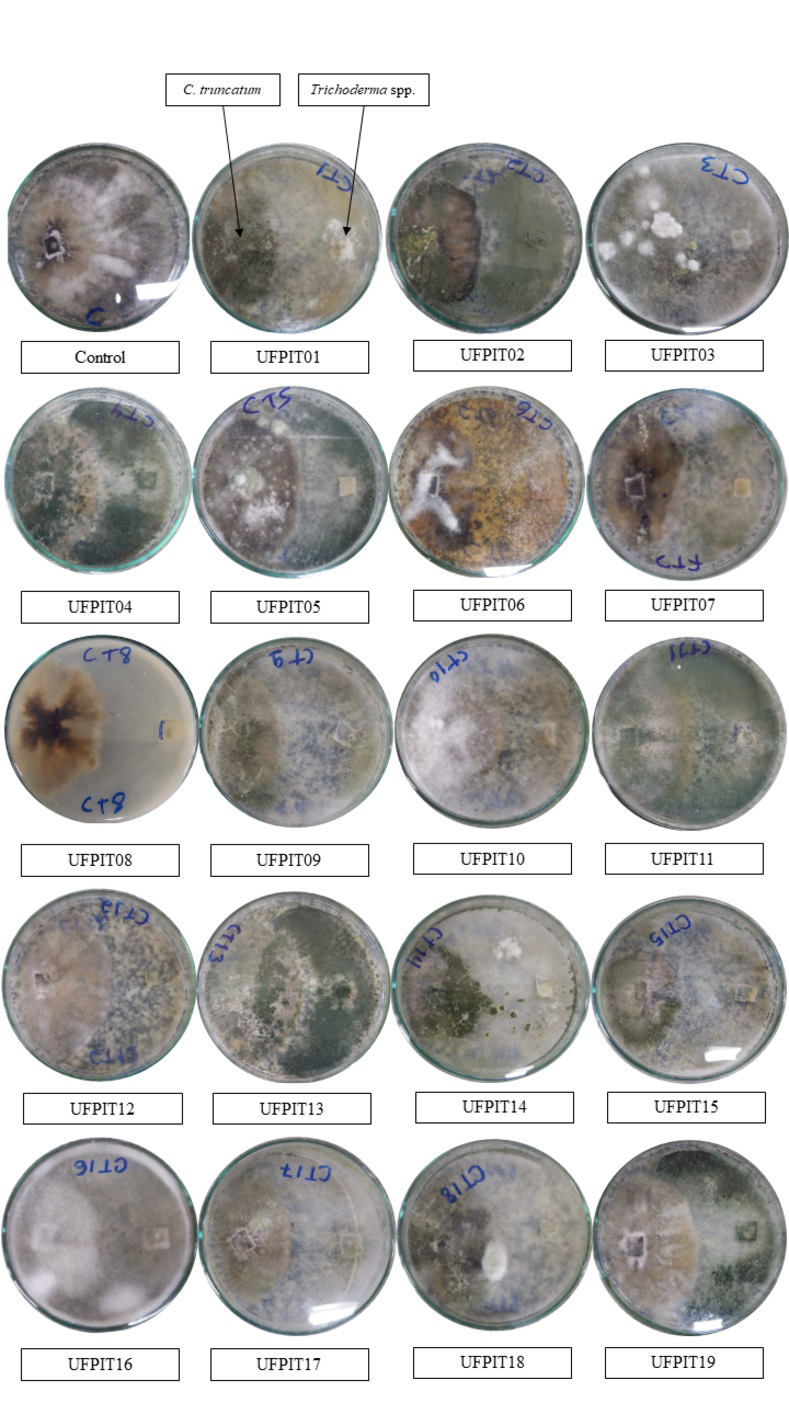

Supplement: S2 Fig — (TIF) [file pone.0265824.s002.tif]

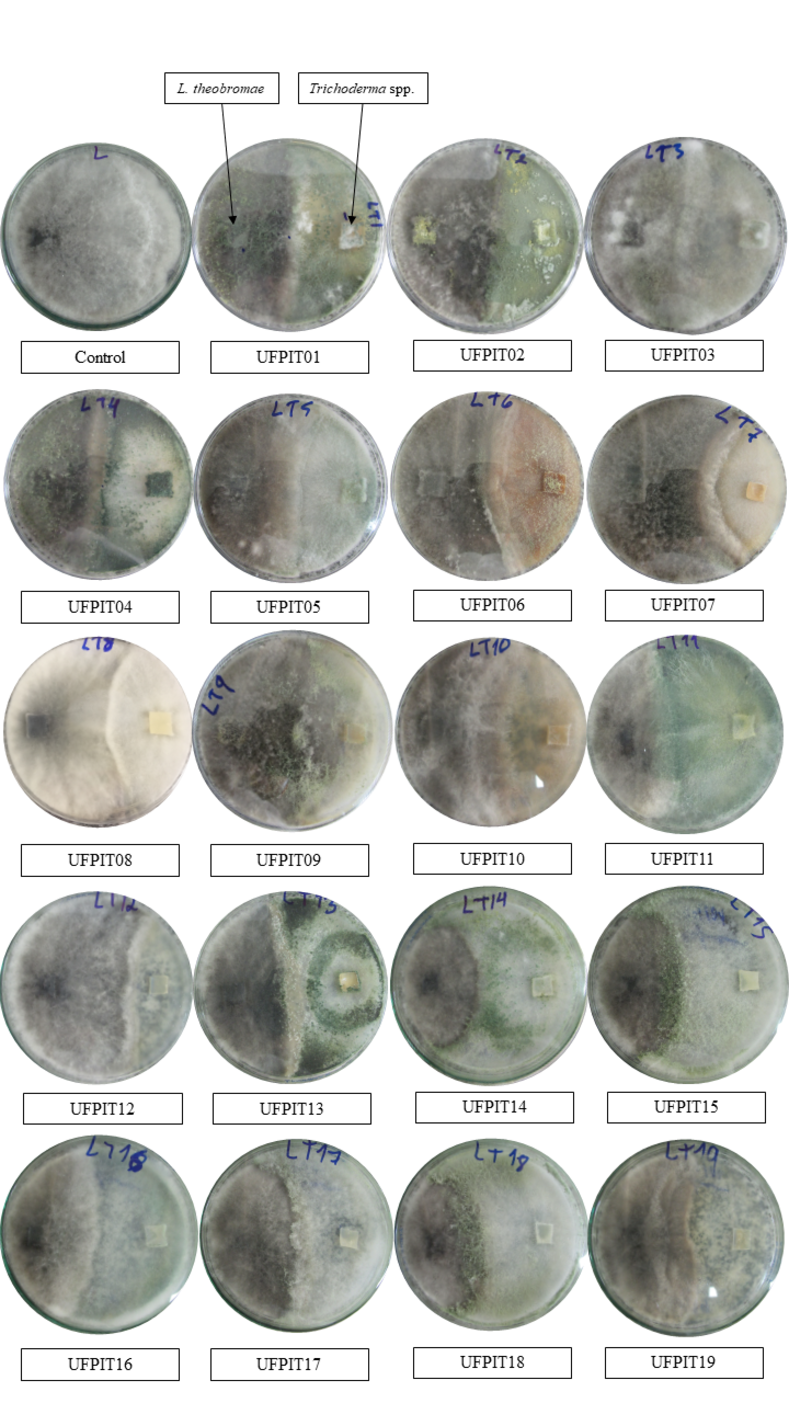

Supplement: S3 Fig — (TIF) [file pone.0265824.s003.tif]

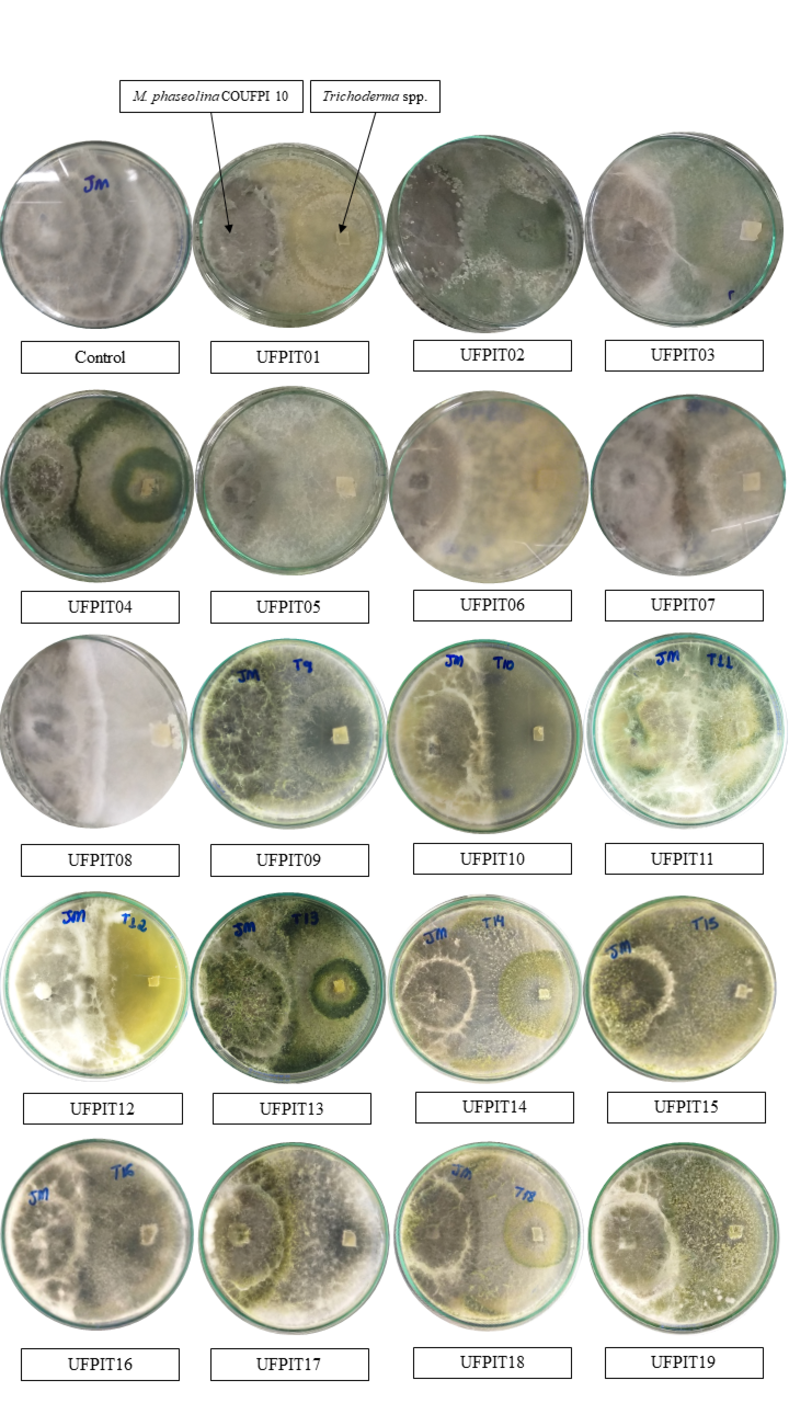

Supplement: S4 Fig — (TIF) [file pone.0265824.s004.tif]

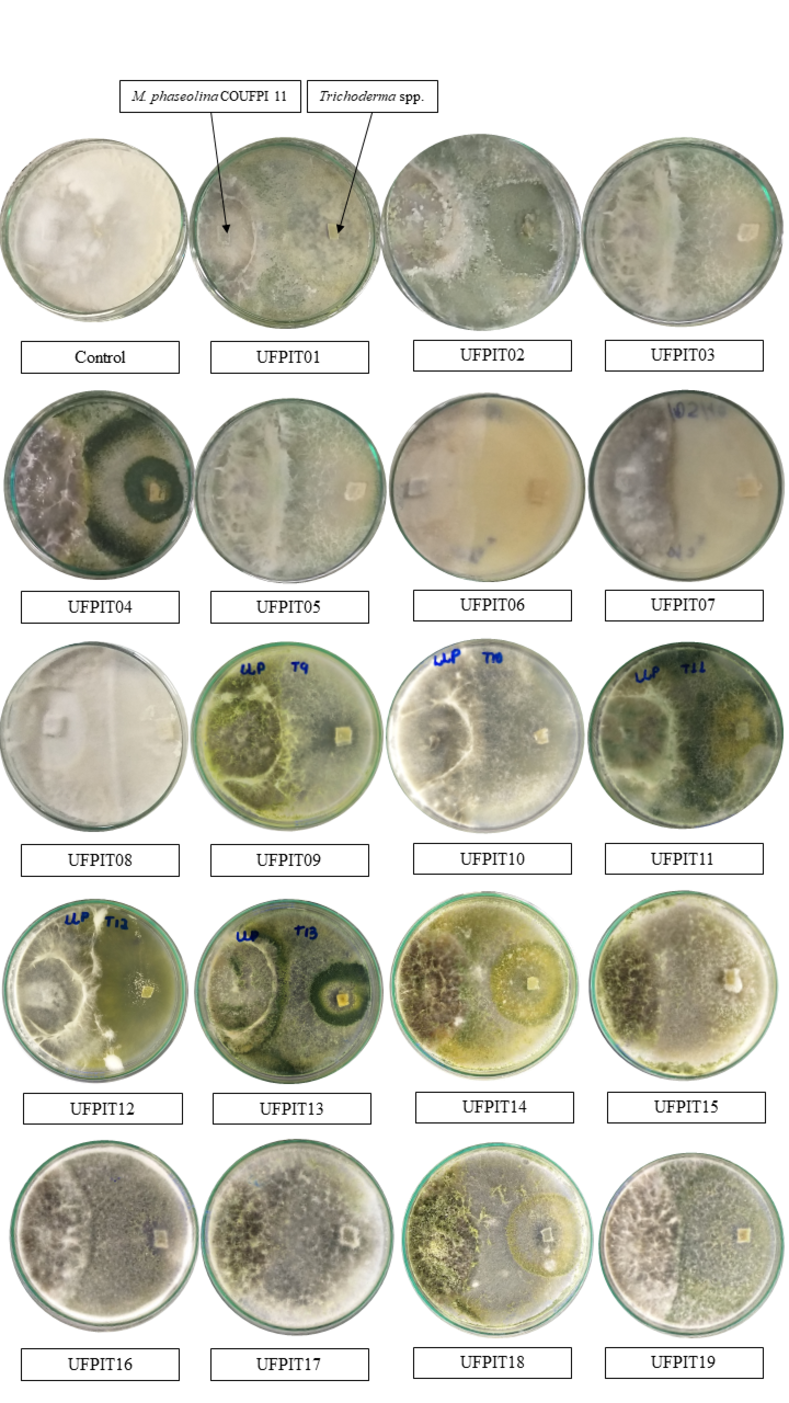

Supplement: S5 Fig — (TIF) [file pone.0265824.s005.tif]

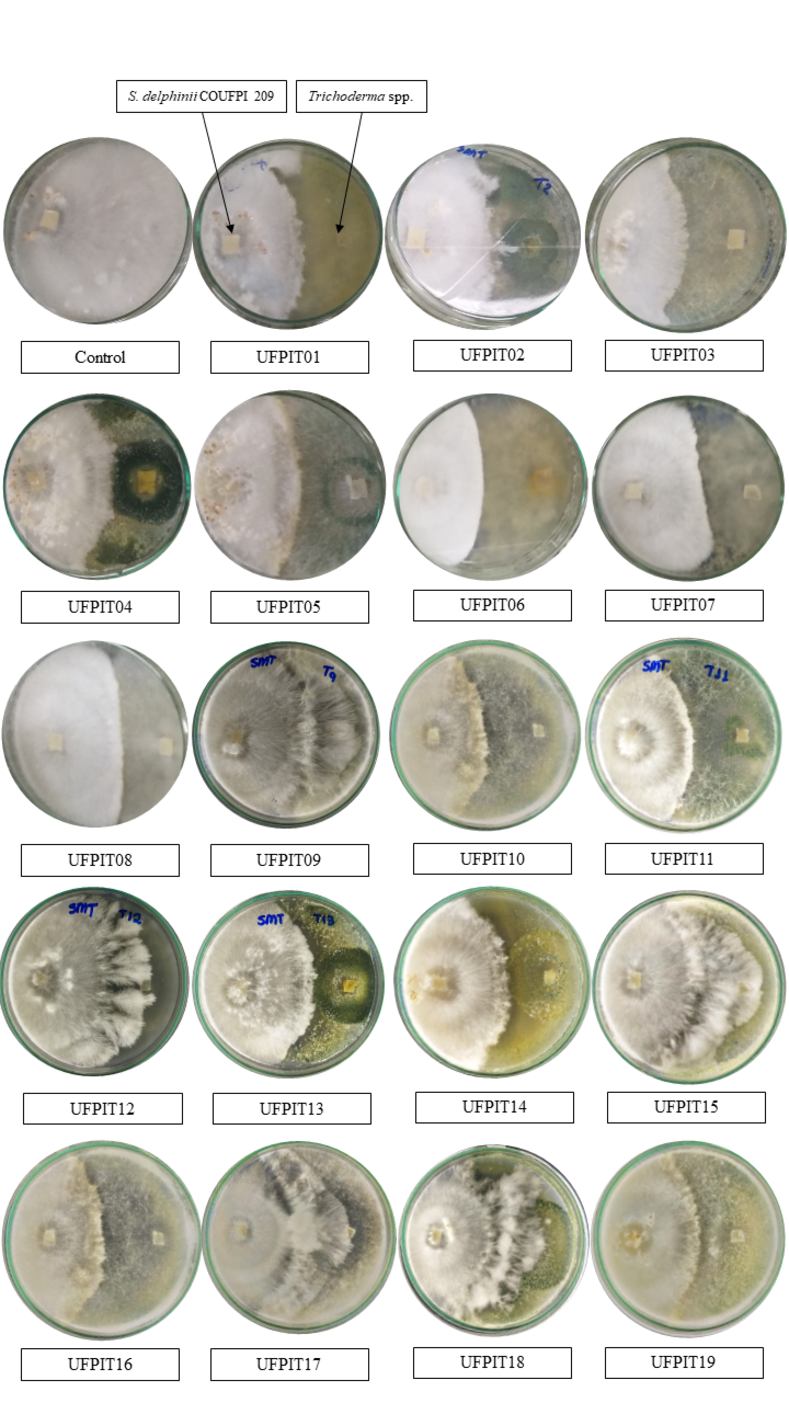

Supplement: S6 Fig — (TIF) [file pone.0265824.s006.tif]

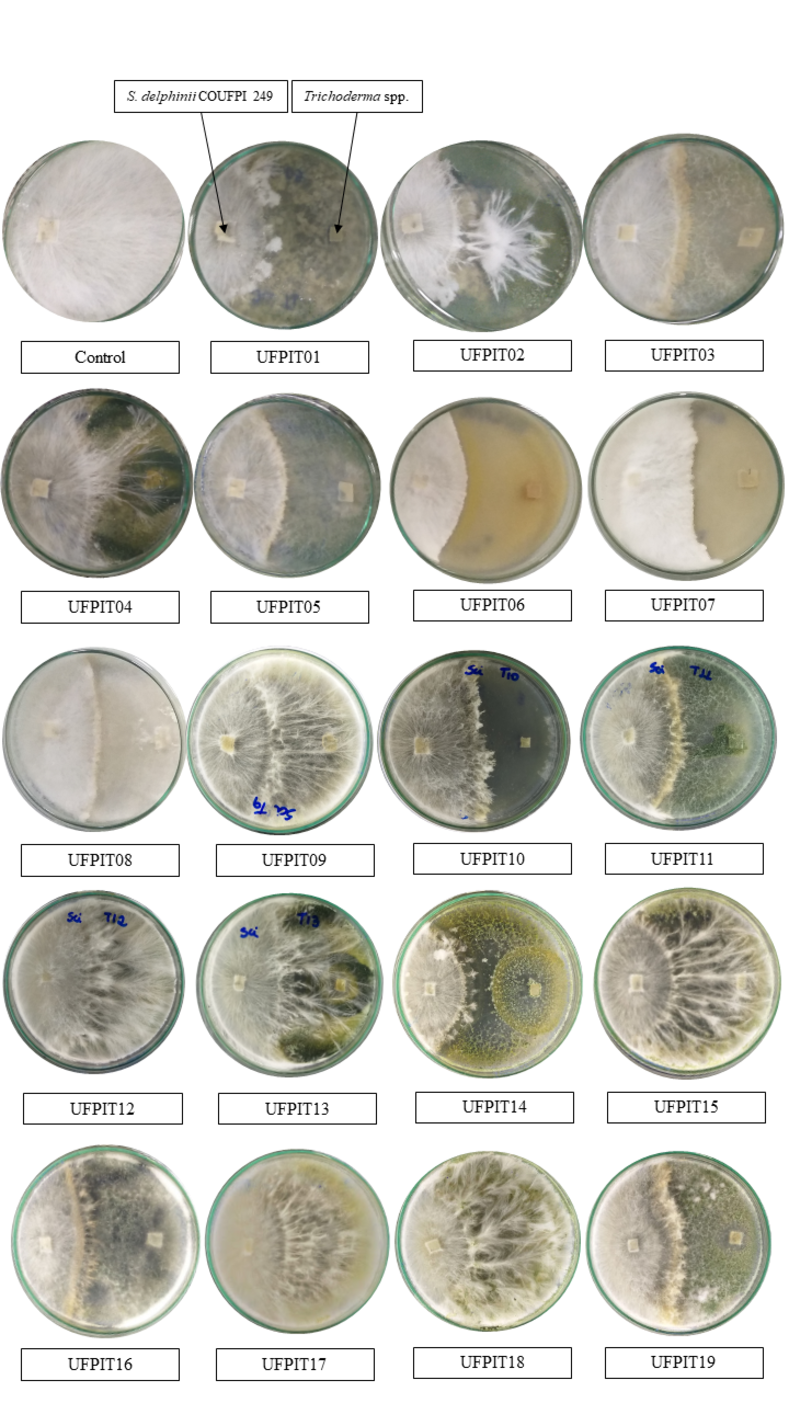

Supplement: S7 Fig — (TIF) [file pone.0265824.s007.tif]

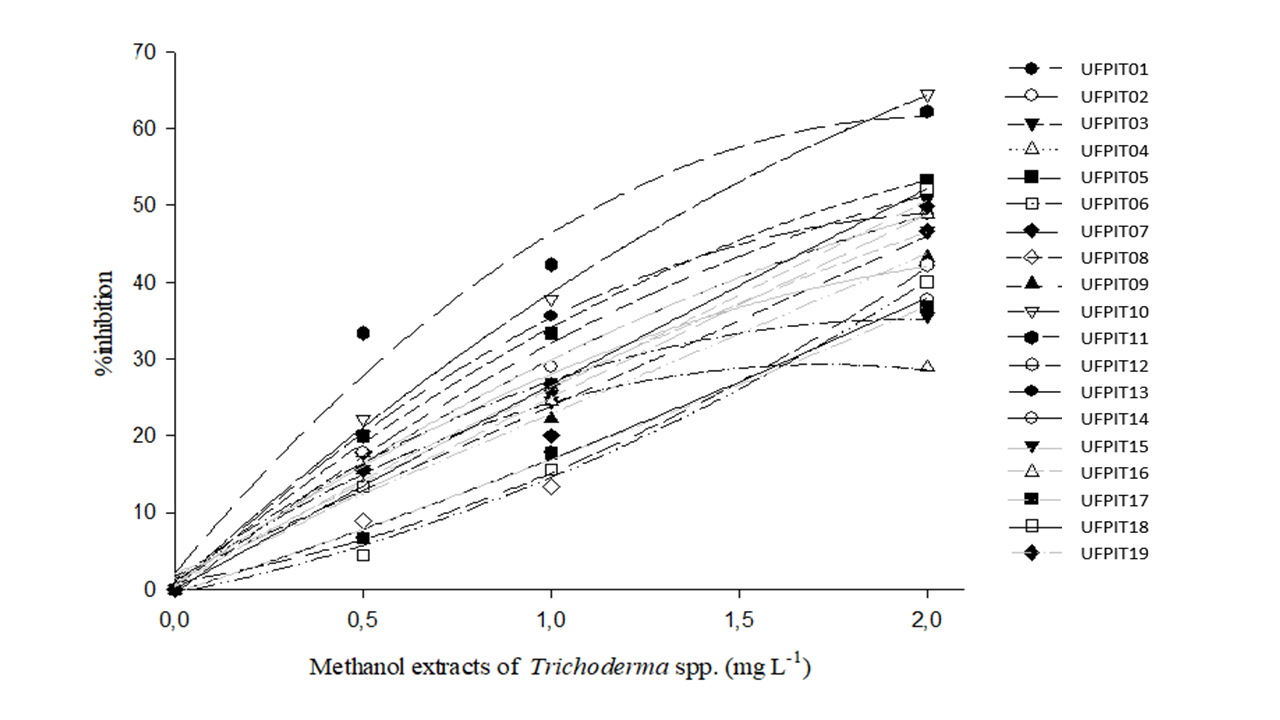

Supplement: S8 Fig — The coefficients of variation (CVs) were 4.65% for the concentration of 0.5 mg mL-1, 8.45% for 1.0 mg mL-1 and 9.36% for 2.0 mg mL-1. (TIF) [file pone.0265824.s008.tif]

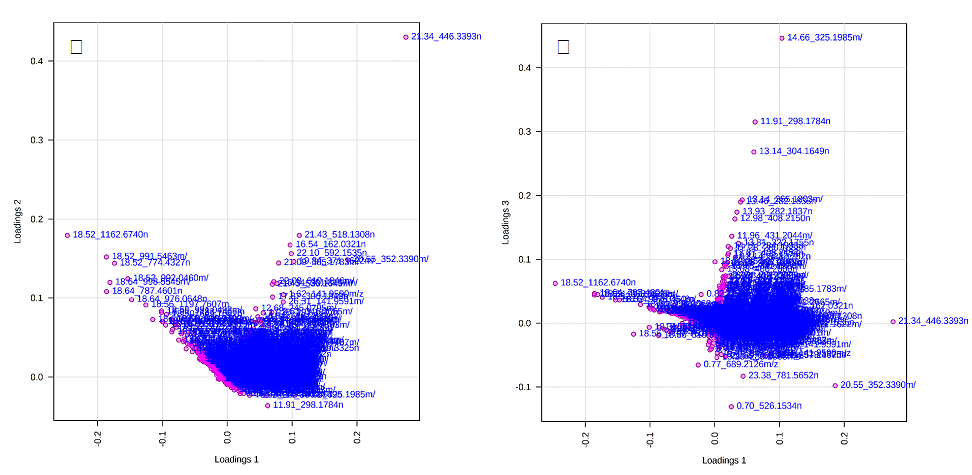

Supplement: S9 Fig — PC1 x PC2 (A) and PC1 x PC3 (B) loading plots of metabolic fingerprints of Trichoderma spp. cultures generated using MetaboAnalyst. Con = Control, UFPIT01 = T1, UFPIT02 = T2, UFPIT03 = T3, UFPIT04 = T4, UFPIT05 = T5, UFPIT06 = T6, UFPIT07 = T7, UFPIT08 = T8, UFPIT09 = T9, UFPIT10 = T10, UFPIT11 = T11, UFPIT12 = T12, UFPIT13 = T13, UFPIT14 = T14, UFPIT15 = T15, UFPIT16 = T16, UFPIT17 = T17, UFPIT18 = T18, and UFPIT19 = T19. (TIF) [file pone.0265824.s009.tif]

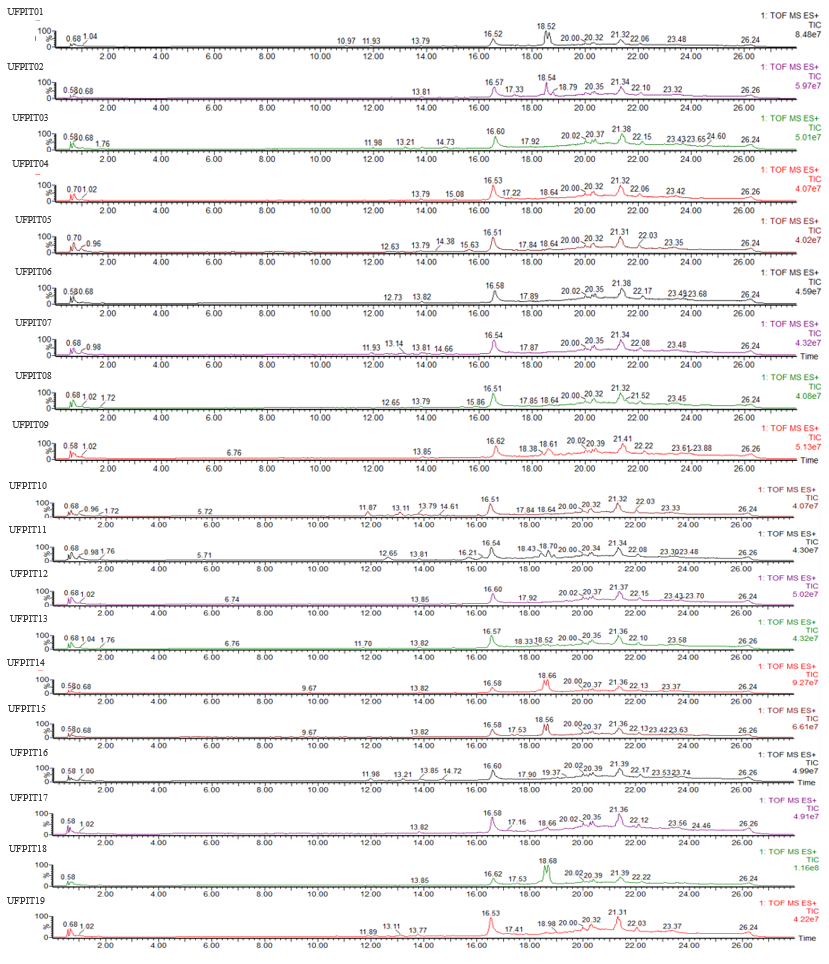

Supplement: S10 Fig — (TIF) [file pone.0265824.s010.tif]

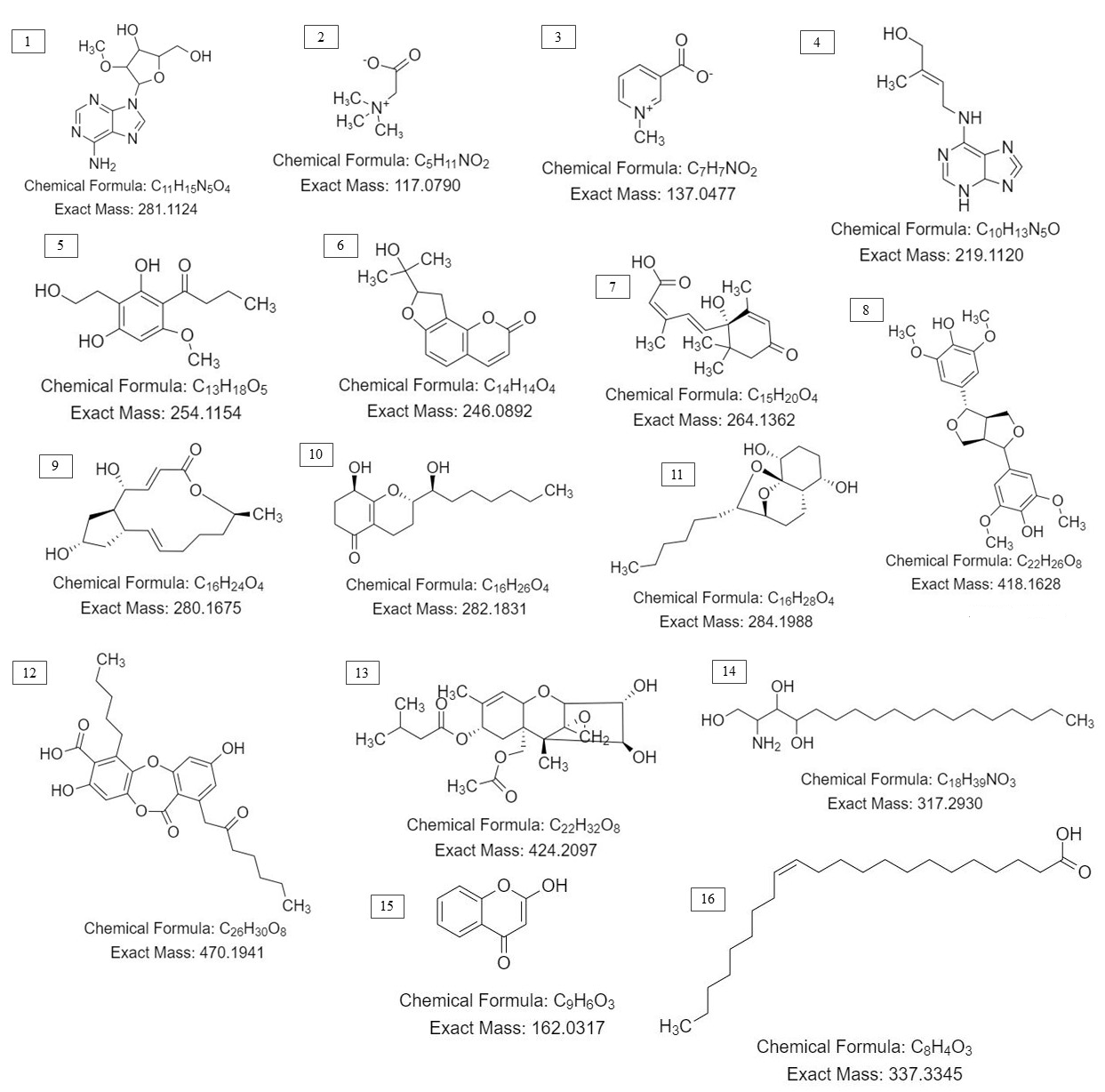

Supplement: S11 Fig — (TIF) [file pone.0265824.s011.tif]
