# Supplementary material for: Endophytic Trichoderma strains isolated from forest species of the Cerrado-Caatinga ecotone are potential biocontrol agents against crop pathogenic fungi
Source: PLoS One. 2022 Apr 15;17(4):e0265824. doi: 10.1371/journal.pone.0265824 (PMC9012399; doi:10.1371/journal.pone.0265824)
Supplement: S1 Table — (DOCX) [file pone.0265824.s012.docx]

**S1 Table.** *Trichoderma* spp. isolates used in this study [29].

| **Isolates** | **Species** | **Isolates** | **Species** |
| --- | --- | --- | --- |
| UFPIT01 | *T. orientale* | UFPIT11 | *Trichoderma* sp. T5 |
| UFPIT02 | *T. longibrachiatum* | UFPIT12 | *T. orientale* |
| UFPIT03 | *T. koningiopsis* | UFPIT13 | *Trichoderma* sp. T6 |
| UFPIT04 | *Trichoderma* sp. T1 | UFPIT14 | *T. orientale* |
| UFPIT05 | *Trichoderma* sp. T2 | UFPIT15 | *T. orientale* |
| UFPIT06 | *Trichoderma* sp. T3 | UFPIT16 | *T. koningiopsis* |
| UFPIT07 | *T. koningiopsis* | UFPIT17 | *T. orientale* |
| UFPIT08 | *Trichoderma* sp. T4 | UFPIT18 | *T. orientale* |
| UFPIT09 | *T. orientale* | UFPIT19 | *T. koningiopsis* |
| UFPIT10 | *T. koningiopsis* |  |  |
