# Supplementary material for: Endophytic Trichoderma strains isolated from forest species of the Cerrado-Caatinga ecotone are potential biocontrol agents against crop pathogenic fungi
Source: PLoS One. 2022 Apr 15;17(4):e0265824. doi: 10.1371/journal.pone.0265824 (PMC9012399; doi:10.1371/journal.pone.0265824)
Supplement: S2 Table — (DOCX) [file pone.0265824.s013.docx]

**S2 Table.** *Trichoderma* spp. isolated from leaves of forest species in an area of Cerrado-Caatinga ecotone [26].

| **Hosts** | **Isolated** |
| --- | --- |
| FABACEAE |  |
| *Cenostigma macrophyllum* Tul. | UFPIT02; UFPIT11; UFPIT15; UFPIT17 |
| *Mimosa tenuiflora* (Willd.) Poir. | UFPIT05; UFPIT10; UFPIT12; UFPIT14 |
| *Bauhinia cheilantha* (Bong.) Steud. | UFPIT06; UFPIT07; UFPIT19 |
| *Diptychandra aurantiaca* (Tul.) Lim. | UFPIT18 |
| *Pityrocarpa moniliformis* Benth. | UFPIT09 |
| MYRTACEAE |  |
| *Myrcia tomentosa* (Aubl.) DC. | UFPIT01; UFPIT03; UFPIT04 |
| COMBRETACEAE |  |
| *Combretum glaucocarpum* Mart. | UFPIT16 |
| *Combretum laxum* Jacq. | UFPIT08 |
| BORAGINACEAE |  |
| *Cordia toqueve* Aubl. | UFPIT13 |
