# Supplementary material for: Endophytic Trichoderma strains isolated from forest species of the Cerrado-Caatinga ecotone are potential biocontrol agents against crop pathogenic fungi
Source: PLoS One. 2022 Apr 15;17(4):e0265824. doi: 10.1371/journal.pone.0265824 (PMC9012399; doi:10.1371/journal.pone.0265824)
Supplement: S3 Table — (DOCX) [file pone.0265824.s014.docx]

**S3 Table.** Pearson's linear correlations between co-culture and crude extract bioassays based on percent inhibition of *Trichoderma* strains against *C. truncatum*.

| **Strains** | | **r** | |
| --- | --- | --- | --- |
| *Trichoderma* spp. | UFPIT04 | -0.65 |  |
|  | UFPIT05 | 0.69 |  |
|  | UFPIT06 | -1.00* |  |
|  | UFPIT08 | 0.97* |  |
|  | UFPIT11 | 0.33 |  |
|  | UFPIT13 | 0.69 |  |
| *T. koningiopsis* | UFPIT03 | -0.56 |  |
|  | UFPIT07 | 0.94 |  |
|  | UFPIT10 | 0.71 |  |
|  | UFPIT16 | 0.30 |  |
|  | UFPIT19 | 0.91 |  |
| *T. orientale* | UFPIT01 | -0.76 |  |
|  | UFPIT09 | -0.65 |  |
|  | UFPIT12 | 0.66 |  |
|  | UFPIT14 | -0.06 |  |
|  | UFPIT15 | 0.29 |  |
|  | UFPIT17 | -0.19 |  |
|  | UFPIT18 | -0.61 |  |
| *T. longibrachiatum* | UFPIT02 | 0.96* |  |

*significant at 5% probability by t test.
